# Characterization of Virulent T4-Like *Acinetobacter baumannii* Bacteriophages DLP1 and DLP2

**DOI:** 10.3390/v15030739

**Published:** 2023-03-13

**Authors:** Danielle L. Peters, Carly M. Davis, Greg Harris, Hongyan Zhou, Philip N. Rather, Sabahudin Hrapovic, Edmond Lam, Jonathan J. Dennis, Wangxue Chen

**Affiliations:** 1 Human Health Therapeutics (HHT) Research Center, National Research Council Canada, Ottawa, ON K1A 0R6, Canada; 2 Department of Biological Sciences, University of Alberta, Edmonton, AB T6G 2E9, Canada; 3 Department of Microbiology and Immunology, Emory University, Atlanta, GA 30322, USA; 4 Research Service, Atlanta VA Medical Center, Decatur, GA 30033, USA; 5 Aquatic and Crop Resource Development (ACRD) Research Center, National Research Council Canada, Montreal, QC H4P 2R2, Canada; 6 Department of Biology, Brock University, St. Catharines, ON L2S 3A1, Canada

**Keywords:** bacteriophage, *Acinetobacter baumannii*, phage therapy, lytic phage, antimicrobial resistance, phage, T4-like phage, *Acinetobacter* phage

## Abstract

The world is currently facing a global health crisis due to the rapid increase in antimicrobial-resistant bacterial infections. One of the most concerning pathogens is *Acinetobacter baumannii,* which is listed as a Priority 1 pathogen by the World Health Organization. This Gram-negative bacterium has many intrinsic antibiotic resistance mechanisms and the ability to quickly acquire new resistance determinants from its environment. A limited number of effective antibiotics against this pathogen complicates the treatment of *A. baumannii* infections. A potential treatment option that is rapidly gaining interest is “phage therapy”, or the clinical application of bacteriophages to selectively kill bacteria. The myoviruses DLP1 and DLP2 (vB_AbaM-DLP_1 and vB_AbaM-DLP_2, respectively) were isolated from sewage samples using a capsule minus variant of *A. baumannii* strain AB5075. Host range analysis of these phages against 107 *A. baumannii* strains shows a limited host range, infecting 15 and 21 for phages DLP1 and DLP2, respectively. Phage DLP1 has a large burst size of 239 PFU/cell, a latency period of 20 min, and virulence index of 0.93. In contrast, DLP2 has a smaller burst size of 24 PFU/cell, a latency period of 20 min, and virulence index of 0.86. Both phages show potential for use as therapeutics to combat *A. baumannii* infections.

## 1. Introduction

Globally, antimicrobial resistance (AMR) has become a major threat to human health, with several bacteria on watch lists due to high levels of antibiotic resistance. Our world is facing a post-antibiotic era and effective novel or alternative treatment options are required. A comprehensive analysis of 204 countries on the impact of AMR estimates that the total global burden of AMR in 2019 was 4.95 million deaths, with 1.27 million caused by bacterial AMR alone [1]. The increase in AMR infections can be attributed to the increased use of antibiotics for secondary bacterial infections and a delay in global action against AMR. The COVID-19 pandemic has further exacerbated the AMR crisis, with a 2022 Centers for Disease Control (CDC) special report describing a 15% increase in resistant nosocomial infections from 2019 to 2020 [2]. 

The World Health Organization has identified *Acinetobacter baumannii*, a Gram-negative bacillus, as a Priority 1 pathogen urgently in need of treatment alternatives due to multidrug-resistant outbreaks [3]. This was further highlighted by the CDC report showing a 78% increase in carbapenem-resistant *Acinetobacter* infections between 2019 and 2020 [2]. *A. baumannii* naturally encodes high levels of innate antibiotic resistance, and is able to quickly acquire new resistance determinants from its environment; thus, the treatment of *A. baumannii* infections are complicated by a limited number of effective antibiotics against this pathogen [4,5]. Thus, alternative therapies must be considered and developed. 

One potential treatment option to address antibiotic-resistant infections is the clinical application of bacteriophages (phages), or viruses that strictly infect bacteria. Phages are ubiquitous in all environments and are isolated from locations in which their host is present; thus, isolation of phages against a wide range of opportunistic pathogens using sewage is very fruitful. Phages are increasingly viewed as a promising treatment option to address the AMR crisis due to their ubiquitous nature, high specificity, low chance of cross resistance with antimicrobials, and reduced impact on the protective gut microbiome [6]. One difficulty with using phages for treatment is their narrow tropism that is often limited to a few strains of a species or species of a genus [7]. Luckily, broad-host range phages have been identified [7,8], and advances in directed evolution approaches and the engineering of host-receptor-binding proteins have shown great success in expanding the host range of target phages [9,10,11]. Several groups have been working to isolate and characterize *A. baumannii* phages for therapeutic use, resulting in a small number of successful case studies using phages against different types of *A. baumannii* infections [12,13,14,15,16,17]. These studies highlight the great therapeutic potential of phages to address infections caused by this problematic pathogen, and many others like it. 

The antibiotic development pipeline has very few antibiotics in an advanced stage of clinical evaluation. Innovative alternatives to antimicrobials, like the therapeutic application of bacteriophages, must be investigated further to address the AMR crisis. In this study, we report the isolation and characterization of two virulent *Acinetobacter* phages vB_AbaM_DLP1 (DLP1) and vB_AbaM_DLP2 (DLP2) which were both found to be members of the T4-like *Lazarusvirus* genus. We show that both DLP1 and DLP2 have large burst sizes, short latent periods, and stability over a range of pH, suggesting that these phages may be good therapeutic candidates. 

## 2. Materials and Methods

### 2.1. Bacteria and Bacteriophages

Bacterial strains, bacteriophages, and plasmids used in this study are listed in Table 1. The appearance of the bacterial colonies selected for overnight cultures were classified as opaque (^O^) or capsule mutant (^cm^).

The *A. baumannii* strains used for phage hunting were AB5075^cm^, 17978^O^, AB0057^O^, AYE^O^, 19606^O^, AB030^cm^, and LAC-4^O^. A single capsule mutant colony, which appears translucent compared to the capsulated cells, was obtained from a streak-for-isolation plate of an AB5075 freezer stock and used to generate a clonal stock of AB5075^cm^. Strains were grown aerobically overnight at 37 °C on Lennox Luria Bertani (LLB) agar plates or in LLB broth with shaking at 200 rpm. Media was supplemented with tetracycline at 5 µg/mL when working with strains carrying the pWH1266 plasmid.

Bacteriophages DLP1 and DLP2 were isolated from an Ottawa, Canada sewage sample using AB5075^cm^ using established protocols [18]. Axenic stocks of DLP1 and DLP2 were generated by three successive rounds of a modified streak-for-isolation technique. Working stocks of DLP1 and DLP2 were propagated using a AB5075^cm^ subculture at an MOI of 100. The lysates were centrifuged at 12,000× *g* for 10 min at 4 °C, filter sterilized with a 0.22 µm PES filter, standardized to 5 × 10^9^ PFU/mL, and stored at 4 °C.

**Table 1:** List of bacterial strains and phages used in this study.

Organisms	Phase and Description	Source
*A. baumannii* AB5075^cm^	Capsule mutant AB5075; host for DLP1 and DLP2	This study
*A. baumannii* AB5075^O^	AB5075; DLP1^R^ and DLP2^R^	[19]
*A. baumannii* AB5075^O^ Δ*wzc*	Clean deletion of *wzc* in AB5075^O^; DLP1^S^ and DLP2^S^	[19]
*A. baumannii* AB5075^O^ Δ*wzc/carO::*MarTc	Clean deletion of *wzc* and Tc1/mariner transposon insertion of *carO* in AB5075^O^; DLP1^S^ and DLP2^R^	This study
*A. baumannii* AB5075^O^ Δ*wzc/carO::*T2*6*	Clean deletion of *wzc* and T26 transposon insertion of *carO* in AB5075^O^; DLP1^S^ and DLP2^R^	This study
pQF1266Blue	Broad-host-range plasmid	[20]
pQF1266Blue + *carO*	Broad-host-range plasmid encoding *carO* gene with native RBS	This study
*A. baumannii* 17978^O^	South Korean isolate ATCC 17978	
*A. baumannii* AB0057^O^	Walter Reed Army Medical Center	[21]
*A. baumannii* AYE^O^	French epidemic strain with high mortality	[22]
*A. baumannii* 19606^O^	ATCC 19606-type strain	[23]
*A. baumannii* AB030^cm^	Hypervirulent XDR Canadian isolate	[24]
*A. baumannii* LAC-4^O^	Hypervirulent XDR American isolate	[25]
*A. baumannii* MRSN 7460	Human wound isolate	[26]
*A. baumannii* MRSN 11669	Human urine isolate	[26]
*A. baumannii* MRSN 15088	Human wound isolate	[26]
*A. baumannii* MRSN 15093	Human wound isolate	[26]
*A. baumannii* MRSN 17493	Human respiratory isolate	[26]
*A. baumannii* MRSN 21681	Human catheter isolate	[26]
*A. baumannii* MRSN 22112	Human blood isolate	[26]
*A. baumannii* MRSN 24603	Human blood isolate	[26]
*A. baumannii* MRSN 30885	Human respiratory isolate	[26]
*A. baumannii* MRSN 31159	Human tissue sample	[26]
*A. baumannii* MRSN 31461	Unknown	[26]
*A. baumannii* MRSN 31915	Unknown	[26]
*A. baumannii* MRSN 31937	Human wound isolate	[26]
*A. baumannii* MRSN 32304	Unknown	[26]
*A. baumannii* MRSN 32875	Unknown	[26]
*A. baumannii* MRSN 32915	Human wound isolate	[26]
*A. baumannii* MRSN 351524	Human blood isolate	[26]
*A. baumannii* MRSN 423159	Human respiratory isolate	[26]
*A. baumannii* MRSN 480561	Human respiratory isolate	[26]
*A. baumannii* MRSN 480622	Human urine isolate	[26]
*A. baumannii* MRSN 489669	Human respiratory isolate	[26]
vB_AbaM_DLP1	Lytic myovirus DLP1	This study
vB_AbaM_DLP2	Lytic myovirus DLP2	This study

^cm^ Capsule mutant; ^O^ Opaque colonies; ^R^ Resistant; ^S^ Sensitive.

### 2.2. Transmission Electron Microscopy (TEM)

For DLP1 TEM analysis, a poly-lysine solution was spotted on a carbon-coated copper grid for 5 min and removed to activate the grid [27,28]. An aliquot of DLP1 was deposited on the grid for 2 min, excess sample was wicked away, and the grid was then stained for 1 min with an aliquot of 2% phosphotungstic acid (PTA). The sample was examined with a JEOL JEM-1400 Flash 120 kV TEM and micrographs were obtained with a Gatan OneView 4 K CMOS camera.

For DLP2 TEM analysis, the grids were freshly glow-discharged using EMS GloQube-D, Dual chamber glow discharge system (Electron Microscopy Sciences, Hatfield, PA, USA) in negative mode with plasma current of 25 mA during 45 s. The grids were floated on a drop of DLP2 lysate for 2 min, rinsed with double distilled water, then stained with 2 % PTA or 1% uranyl acetate for 60 s. A HITACHI H-7500 (Japan) equipped with bottom-mounted AMT NanoSprint 12 MP camera operating at 80 kV in high-contrast mode was used to obtain the micrographs.

The dimensions of ten phage capsids and tails were measured for DLP1 and DLP2 using ImageJ v1.53 (National Institutes of Health, Bethesda, MD, USA).

### 2.3. Host Range and Efficiency of Plating (EOP)

Phages DLP1 and DLP2 were screened against a panel of 107 strains of *A. baumannii.* Briefly, two 10 µL aliquots of DLP1 or DLP2 (5 × 10^9^ PFU/mL) were spot-plated onto each strain, and incubated overnight at 37 °C. Strains that showed clearing were selected for efficiency of plating analysis.

Overnight cultures of phage-sensitive strains were used to make double layer plates. A tenfold dilution series of each phage stock (2.5 × 10^9^ PFU/mL) was spotted in triplicate 5 µL aliquots onto each phage-sensitive strain and incubated at 37 °C overnight. The plates were assessed for plaque formation or clearing compared to SM controls.

### 2.4. One-Step Growth Curves

A one-step growth curve of DLP1 and DLP2 was generated on *A. baumannii* strain AB5075^cm^ as previously described with minor modifications [29]. Overnight cultures of AB5075^cm^ were subcultured 1:100 and grown for ~80 min to an OD_600_ of 0.2. Each phage was added to individual subcultures at a multiplicity of infection (MOI) of 0.001 and incubated at 37 °C with shaking at 200 rpm. At 5 min post-infection, the infected cultures were diluted 1:100. Triplicate aliquots of the infection were removed at various time points and immediately plated. Phage titers were determined by either spot-plating a 5 µL dilution series on TALLB double layer plates impregnated with AB5075^cm^, or mixing 100 µL dilution series with 100 µL of overnight AB5075^cm^ culture and 3 mL of TALLB for double agar overlay assay plating. Burst size was calculated with the formula “burst size = P/I” where P is the maximum number of phages after lysis, and I is the number of phages initially added to the culture. Resulting data from three biological and technical replicates were analyzed using GraphPad Prism 9 (GraphPad Software Inc., San Diego, CA, USA).

### 2.5. Virulence Assay of DLP1 and DLP2 against AB5075^cm^

Kill curves of DLP1 and DLP2 against AB5075^cm^ were performed as previously described [30] with minor modifications and performed in biological and technical triplicates. Briefly, 100 µL aliquots of a 2 h subculture of AB5075^cm^ were mixed with 100 µL of DLP1 or DLP2 at various MOIs in a 96 well plate. The plate was incubated with a LogPhase 600 spectrophotometer (Agilent, Santa Clara, CA, USA) at 37 °C with 800 rpm orbital shaking. OD_600_ was obtained every hour for 24 h.

The virulence index was calculated during exponential growth, with the data cut-off at 8 h. Local virulence (V_L_) was calculated by dividing the area under the curve (AUC) for each MOI by the AUC of the AB5075^cm^ control. This value was subtracted from 1 to determine the V_L_ for each MOI. The global virulence index (V_i_) was calculated by averaging the AUC obtained from plotting the AUC of each MOI V_L_ against log_10_ MOI. Data were collected in technical and biological triplicates, then analyzed using GraphPad Prism 9.

### 2.6. Phage pH Stability Assay

A 100 µL aliquot of AB5075^cm^ overnight was inoculated into 3 mL molten (~50 °C) TALLB and poured onto a dry LLB plate and allowed to dry for 1 h. Standardized stocks of DLP1 and DLP2 (2.5 × 10^9^ PFU/mL) were diluted 1:100 into SM adjusted to various pH values (3, 5, 7, 9, and 11) or into an SM control (pH ~7.5). Samples were incubated at room temperature for 1 h, and aliquots of each sample were serially diluted, and spot-plated (5 µL) on the TALLB plates. Spots were allowed to absorb, and plates were incubated overnight at 37 °C. Plates were then scored for PFU. The experiment was completed in biological and technical triplicate.

### 2.7. Phage Temperature Stability Assay

A 100 µL aliquot of AB5075^cm^ overnight was inoculated into 3 mL molten (50 °C) TALLB and poured onto a dry LLB plate and dried for 1 h. Then, 50 µL aliquots of standardized stock (2.5 × 10^9^ PFU/mL) of DLP1 and DLP2 were dispensed into low-bind PCR tubes and incubated for 1 h at 4, 30, 40, 50, 60, 70, 80, and 90 °C. Samples were cooled to RT and aliquots were serially diluted and spot-plated (5 µL) on the square plates. Spots were allowed to absorb, and plates were incubated overnight at 37 °C. Plates were then scored for PFU.

### 2.8. Biofilm-Inhibition Assessment of DLP1 and DLP2

An overnight culture of AB5075^cm^ was subcultured 1:100 into LLB and grown for 1.5 h at 37 °C with shaking at 200 rpm. Wells of a tissue culture-treated, clear, flat-bottom 96 well plate were filled with 180 µL of LLB + 1% dextrose, then inoculated with 10 µL of the subculture, and treated with 10 µL of standardized phage suspension (MOI 10, 1, 0.1), or SM for control wells. The subculture was serially diluted with PBS and spot-plated for colony counts of the final inoculum. Serial dilutions of the phage suspensions were spot-plated to confirm their titer at time zero.

Plates were incubated at 37 °C for 24 h, then plate contents were removed and 300 µL of 0.9% NaCl was used to gently wash the plate twice. The plate was dried at 50 °C for 1 h and stained with 250 µL 0.1% crystal violet for 15 min. The plate was washed three times with 300 µL distilled H_2_O and air dried for 30 min at RT. The wells were then de-stained for 5 min with 250 µL 30% acetic acid, gently mixed 10 times, and a 150 µL aliquot was removed from each well to a new 96-well plate. The optical density (600 nm) was recorded and analyzed in GraphPad Prism. The experiment was repeated in biological triplicate and technical quadruplicate.

### 2.9. Identification of DLP2 Receptor

*A. baumannii* AB5075 Δ*wzc* [31] was mutagenized with a derivative of the mariner transposon MAR2XT7 [32], where a tetracycline resistance gene was inserted into the gentamicin resistance gene, resulting in MarTc. Using natural transformation, *E. coli* SM10 containing the modified mariner element on the suicide plasmid pMAR2XT7 was grown to mid-log phase and mixed with an equal volume of mid-log phase AB5075 Δ*wzc*. Cell mixtures were spotted on a dried LLB plate and incubated for 5 h. Cells were then resuspended in LLB and plated on LLB agar plates containing ciprofloxacin (1 mg/mL) and tetracycline (5 mg/mL). A pool of approximately 10,000 colonies was then plated on an LLB agar plate and flooded with a suspension of DLP1 or DLP2 phage. The MarTc insertion site was identified by cloning partial Sau3A fragments into pBC.SK and selecting for tetracycline resistance encoded by MarTc. A primer reading outward from MarTc into the chromosome was used to identify the disrupted gene.

To independently confirm the phenotype of the mutant, DNA from the ordered *A. baumannii* library [33] was moved into the *A. baumannii* AB5075 Δ*wzc* strain by natural transformation as described above. Briefly, a stationary phase culture of strain AB02742 from the ordered library was filter-sterilized, and the supernatant was mixed with an equal volume of early log phase AB5075 Δ*wzc* cells. The mixture was spotted onto a well-dried LLB plate and incubated for 6.5 h at 37 °C. Cells were then resuspended in LLB and plated on 5 mg/mL tetracycline. Verification of the mutation was carried out by PCR.

### 2.10. Complementation of the carO Mutant

A wild-type copy of the *carO* gene with its native ribosome-binding site was generated by PCR amplification using Phusion DNA polymerase and the primers: carO.for AAATTAAAAACAGAGCCTTTTC and carO.rev AAGCTCGTTTTTATGCTTTATTACC. This PCR-derived fragment was cloned into the *Sma*I site of pQF1266Blue [20], where transcription was driven from a strong endogenous promoter. Plasmid pQF1266Blue alone and pQF1266Blue containing the *carO* gene were moved into *A. baumannii Δwzc carO::T26* by electroporation and selection on LLB plates containing 150 mg/mL hygromycin. Infection efficiency of DLP1 and DLP2 on both *carO* transposon mutants and the *Δwzc carO::T26*-complemented strain compared to the AB5075 *Δwzc* host was completed in biological duplicate.

### 2.11. Phage Concentration, DNA Isolation, Sequencing, and RFLP Analysis

Genomic DNA (gDNA) was isolated from high-titer DLP1 and DLP2 stocks (>10^9^ PFU/mL). A 25 µL aliquot of 1 M MgCl_2_ and 5 µL aliquot of 1 M CaCl_2_ was added to each lysate. A 4 µL aliquot of DNase I (2000 U/mL) and a 10 µL aliquot of RNase A (100 mg/mL) were added to the lysate and incubated at RT for 1 h. Following incubation, 4 µL of 1 M NaCl and 1 g of PEG 6000 was added to each sample and vortexed until the PEG dissolved, then stored at 4 °C overnight. The samples were then centrifuged at 20,000× *g* for 30 min at 4 °C, and the resulting pellets were resuspended in an equal volume of SM. A final centrifugation of 5000× *g* for 10 min at 4 °C was performed and the supernatant recovered for DNA extraction.

A 970 µL aliquot of each concentrated phage sample was mixed with 25 μL of 100 mM MgCl_2_ and 5 µL of 100 mM CaCl_2_. A 1 µL aliquot of DNase I and RNase A was added to each sample, inverted to mix, and incubated at 37 °C for 1 h with 300 rpm shaking. Following incubation, 40 µL of 0.5 M EDTA was added to each tube and vortexed for 10 s. A 2.5 µL aliquot of proteinase K (20 mg/mL) and 50 µL aliquot of 10% SDS (*w/v*) were added to each tube and incubated at 55 °C for 1 h with 300 rpm shaking. An equal volume of Phenol:Chloroform:Isoamyl alcohol (PCI) was mixed with the lysate and transferred to PhaseLok tubes for centrifugation at 16,000× *g* for 10 min at RT. The PCI extraction was performed twice, followed by an equal volume chloroform extraction to remove residual PCI. The resulting DNA was pelleted with ethanol and 0.3 M sodium acetate following an overnight −20 °C incubation. The DNA pellets were washed twice with ice-cold 70% ethanol, air dried, and resuspended in nuclease-free water.

The purity and concentrations of resulting DNA were checked with a NanoDrop ND-1000 spectrophotometer (Thermo Scientific, Waltham, MA, USA) and run on a 0.7% TAE gel to check gDNA integrity. DLP1 and DLP2 gDNA were mechanically sheared with Adaptive Focused Acoustics (Covaris, Woburn, MA, USA), and the libraries were prepared with a NEB Ultra II DNA Library prep kit (NEB, Ipswich, MA, USA). Paired-end reads were sequenced on Miniseq 300 cycle mid output flow cell (Illumina, Inc., San Diego, CA, USA).

Restriction fragment length polymorphism (RFLP) analysis [1,16,18,34,35,36,37,38,39,40,41,42] was performed on 1 µg of DLP1, DLP2, and *Escherichia* phage Lambda (control) gDNA. The DNA was subjected to a panel of 16 restriction enzymes: *Eco*RI, *Bam*HI, *Bg*III, *Eco*321, *Hind*III, *Kpn*I, *Sma*I, *Pst*I, *Sa*II, *Nde*I, *Not*I, *Xho*l, *Xba*I, *Msp*I, *Hpa*I, and *Pae*I (FastDigest, Thermo Scientific, Waltham, MA, USA). Reactions and uncut controls were separated on a 1.2% agarose gel in 1x TAE (pH 8.0) and stained with GelRed (Biotium, Freemont, CA, USA) for 15 min prior to imaging on a ChemiDoc system (Bio-Rad, Hercules, CA, USA).

### 2.12. Bioinformatics

Reads were assessed with FastQC v.0.11.9 and paired in Geneious Prime v.2022.2.2 with the following insert sizes: DLP1, 166 bp; DLP2, 217 bp. The paired reads were trimmed with BBDuk v.38.84 for 158 adapter sequences, minimum Q20 ends, and short reads (<50 bp) were discarded. The trimming and filtering of DLP1 reads retained 93.9% of the 3,022,060 reads with a Q30 of 99.0%. The trimming and filtering of DLP2 reads kept 97.3% of the 3,506,104 reads with a Q30 of 98.9%. The trimmed reads underwent de novo SPAdes v.3.15.2 [43] assembly using the following parameters: multicell, error correct and assemble, and careful mode with 5% of the reads for both DLP1 and DLP2. Reads were re-aligned to each contig using Bowtie2 v.2.4.5 [44] and the following parameters: end to end, medium sensitivity, with only the best match reported.

The DLP1 and DLP2 genomes were analyzed with PhageTerm [45] in Galaxy [46] using the SPAdes contigs and trimmed reads as input. Genes were called using PHANOTATE [47], Glimmer3 v1.5 [48], and Geneious ORF finder, then manually curated. The tRNA were identified using tRNAscan-SE v.2.0.5 [49]. Phage promoters and terminators were determined using PhagePromoter [50] and TransTermHP v.19.1.0.0 [51]. Protein functions were assigned based on the InterProScan of protein domains with the following applications run: CDD, Coils, Gene3d, HAMAP, MobiDB-Lite, Panther, PfamA, Phobius, PIRSF, PRINTS, PrositePatterns, PrositeProfiles, SFLD, SignalP, SignalP_EUK, SignalP_GRAM_NEGATIVE, SMART, SuperFamily, TIGRFAM, and TMHMM. Geneious Annotate by BLAST was used to further annotate the genome using the nucleotide collection (nr/nt) with five hits maximum and a similarity cut-off of 75%. The BLOSUM62 matrix was used with 11 1 gap cost, word size of 6, and max E-value of 0.05.

The average nucleotide identity (ANI) analysis of DLP1 and DLP2 was performed using the ANI Calculator (http://enve-omics.ce.gatech.edu/ani/, accessed on 20 September 2022) [52] and the following options: min length, 700 bp; min ID, 70%; min alignments, 50; window size, 1000 bp; and step size, 200 bp. Genomic comparison of DLP1 and DLP2 was performed using LASTZ v.1.04.15 [53,54] using DLP1 as target and DLP2 forward and reverse strands compared with the following parameters: step length, 20; seed pattern, 12 of 19; perform chaining; perform gapped alignment; search both strands; HSP threshold limit, 3000; and gapped threshold limit, 3000. Protein alignments were performed using Clustal Omega v.1.2.3 [55], with the following parameters: eight refinement iterations, fast clustering for initial guide tree (mBed), full distance matrix for refinement iteration guide tree, cluster size of 100 for mBed guide trees. PHYML v.3.3.20180621 was used for tree building with 100 boostrap branch supports and 4 substitution rate categories, optimizing for topology/length/rate [56].

Further analysis on proteins of interest was completed using PHYRE2 [57] batch submit and the EBI Protein Similarity Search tool [58] against the following databases: uniprotkb_swissprot, AlphaFold (afdb) [59], uniprotkb_pdb. Whole genome alignments were completed using MAFFT [60] and Clinker [61]. Genomes were submitted to NCBI with the accession numbers OP946501 and OP946502 for DLP1 and DLP2, respectively.

### 2.13. Statistical Analysis

Data collected were analyzed in GraphPad Prism 9. A Kruskal–Wallis test with Dunn’s multiple comparisons test was performed on the data to determine statistical significance. The experiment was completed in biological and technical triplicate, unless otherwise stated. *p* < 0.05 is considered statistically significant.

## 3. Results

### 3.1. Phage Isolation, Morphology, and Host Range

#### 3.1.1. Phage Isolation and Morphology

The myovirus phages DLP1 and DLP2 (vB_AbaM-DLP_1 and vB_AbaM-DLP_2, respectively) were isolated from two influent sewage samples collected in the Ottawa area using the *A. baumannii* host strain AB5075^cm^, a capsule mutant of AB5075. The plaques produced by DLP1 are clear with a diameter of 0.5 to 1 mm. A halo is present around the plaques after an overnight incubation at 37 °C, and it continues to expand outward if left at RT over days, suggesting the presence of a diffusible factor. DLP2 also produces clear plaques with a diameter of 0.5 to 1 mm, but no halo is present or develops over the course of RT incubation.

**Figure 1:**
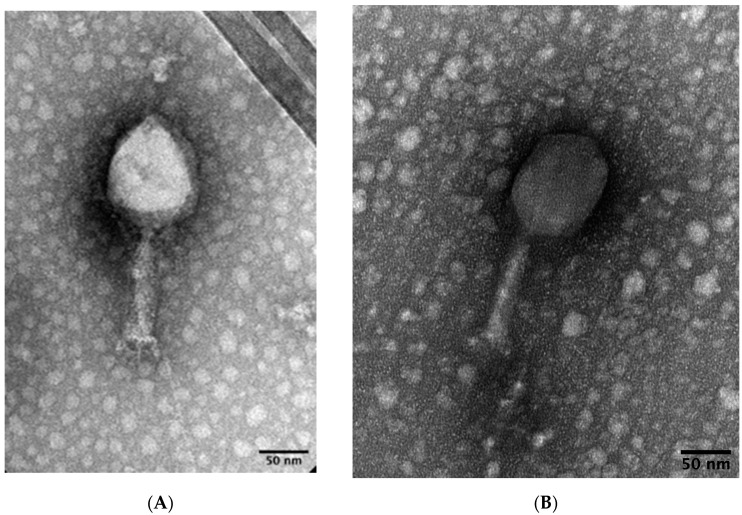
Transmission electron micrographs of two myoviruses. High titer stocks of liquid propagated DLP1 (**A**) and DLP2 (**B**) lysates were stained with 2% PTA or 1% uranyl acetate, respectively, on a copper grid at 120 or 80 kV, respectively. Prolate capsids, contractile tails, tail fibers, and base plates indicate that DLP1 and DLP2 are myoviruses.

Transmission electron microscopy was used to determine morphology of phages DLP1 and DLP2. Both phages are classified as members of the A2 (myovirus) morphotype based on their prolate heads and contractile tails with tail fibers (Figure 1). The average measurement and standard deviation of ten intact DLP1 virions micrographs reveals a tail length and width of 109 ± 2.6 and 21 ± 0.9 nm, respectively, while the DLP1 capsid length and width is 112 ± 3.0 and 88 ± 3.9 nm, respectively (Figure 1A). In contrast, the average measurement of intact DLP2 virions for tail length and width is 116 ± 1.8 and 23.6 ± 1.0 nm, respectively, while the average DLP2 capsid length and width measures 113 ± 2.2 and 84 ± 1.3 nm, respectively (Figure 1B).

#### 3.1.2. Host Range and Efficiency of Plating (EOP)

**Figure 2:**
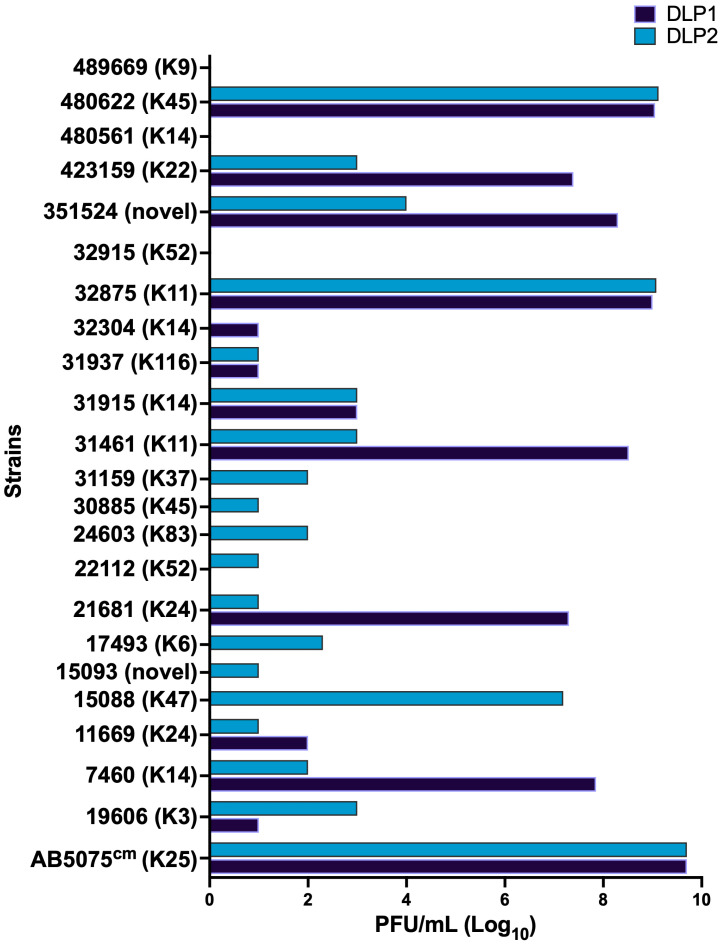
Efficiency of plating for DLP1 and DLP2 susceptible strains with K-type listed in brackets. A dilution series of standardized DLP1 and DLP2 stock (2.5 × 10^9^ PFU/mL) was spot-plated on each host and scored for level of infectivity. An EOP of zero indicates decreased turbidity on the phage spot-plates at the highest concentration, but no plaquing is observed. Data represent the mean of a technical triplicate.

Host range analysis of these phages against 107 *A. baumannii* strains shows a limited host range, infecting 15 and 21 strains for phages DLP1 and DLP2, respectively. The susceptible strains were further examined for productive phage infection by determining EOP of each phage on their sensitive strains. A serial dilution of phage lysate was spotted on the sensitive strain and compared to the main host AB5075^cm^. EOP experiments with the sensitive strain panel show that DLP1 tends to have a higher EOP in the strains it infects compared to DLP2 (Figure 2). The BEI strains 480622, 423159, 351524, 32875, 31461, 21681, and 7460 are highly susceptible to DLP1 and show good EOP profiles compared to the host strain AB5075^cm^ (Figure 2). On the other hand, DLP2 infects more strains than DLP1, but with less efficiency overall. The BEI strains that DLP2 can infect with high efficacy are 480622, 32875, and 15088 (Figure 2). Interestingly, both phages are capable of infecting capsulated ATCC 19606, which is K-type 3. The only other strain with capsule-type 3 screened for susceptibility was ATCC 17978, which is resistant to DLP1 and DLP2.

### 3.2. Burst Size, Virulence and Stability of DLP1 and DLP2

#### 3.2.1. Burst Size

**Figure 3:**
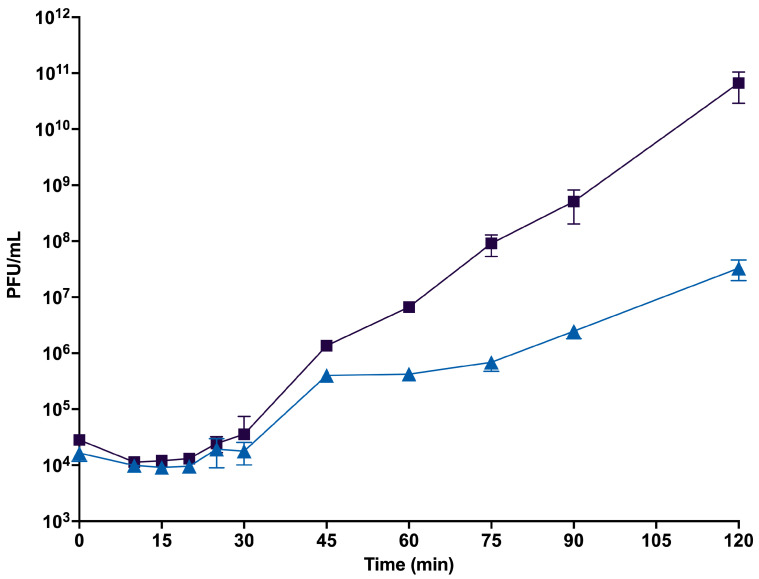
Burst size of phages DLP1 (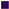) and DLP2 (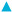) on host strain AB5075^cm^. The mean of biological and technical triplicates represented at each time point and error bars represent standard deviation.

The burst size of DLP1 and DLP2 shows that both phages have a latent period of approximately 20 min, and a cycle completion of approximately 60 min (Figure 3). The burst size for DLP1 was calculated to be 236 PFU, while the burst for DLP2 was a log lower at 26 PFU.

#### 3.2.2. AB5075^cm^ Suppression and Virulence Curves of DLP1 and DLP2

**Figure 4:**
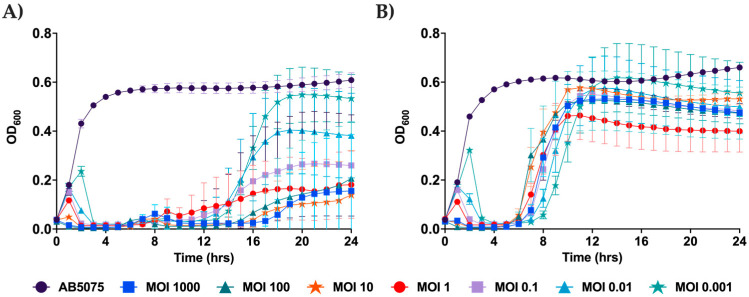
Growth suppression analysis of phages DLP1 (**A**) and DLP2 (**B**) against strain AB5075^cm^ over 24 h. The multiplicity of infection (MOI) range was studied from 0.001 to 1000 for each phage. The mean and standard deviation are represented from three biological and technical triplicate experiments.

The ability of a phage to suppress the growth of a host strain over time is very important for therapeutic applications; thus, a growth suppression analysis of DLP1 and DLP2 against AB5075^cm^ was completed. There are distinct differences in the ability of DLP1 and DLP2 to suppress growth over time at various MOIs (Figure 4). DLP1 is capable of suppressing the growth of AB5075^cm^ across all MOIs; an MOI of 1000 suppresses growth of the bacterium for 17 h and maintains a threefold reduction in optical density compared to the untreated control at 24 h (Figure 4A), whereas the lowest MOI of DLP1 tested (0.001) suppresses the growth of AB5075^cm^ for up to 13 h. By 18 h, the optical density is similar to the untreated control group (Figure 4A). In contrast, DLP2 is less efficient at suppressing growth over time, with all MOIs displaying OD increases beginning at 6 h and MOIs 0.001 and 10 approaching untreated OD levels by 11 h (Figure 4B). The optimal MOI with DLP2 is 1 compared to the untreated control (Figure 4B).

**Figure 5:**
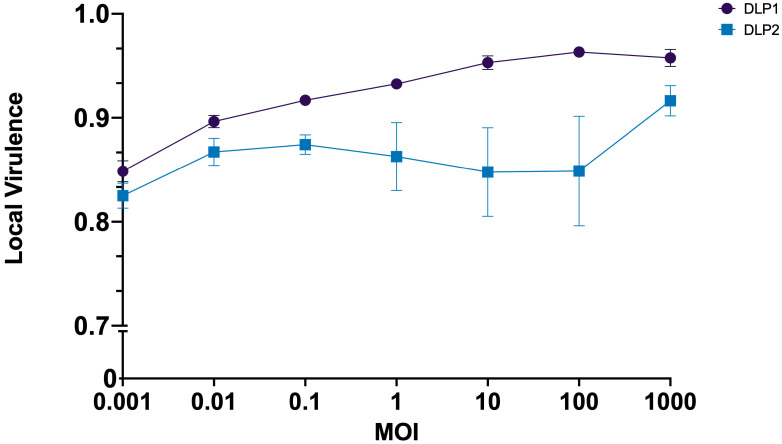
Virulence index of phages DLP1 (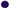) and DLP2 (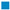) against *A. baumannii* AB5075^cm^. The OD_600_ of kill curves was measured across known MOIs (Figure 4) and virulence curves were generated as described in the methods section. A virulence index of 1 signifies theoretical maximum virulence. The data represent mean and standard deviation of technical and biological triplicate experiments.

The data collected from the growth suppression experiments were further analyzed to determine the local virulence (V_L_) for each phage at each MOI against AB5075^cm^ (Figure 5). The overall virulence indexes (V_i_) for DLP1 and DLP2 are 0.93 and 0.86, respectively, although DLP1 shows the highest V_L_ at an MOI of 100 (0.96). Phage DLP2 shows high V_L_ variability at MOIs of 10 and 100, but the variability greatly reduces at MOI 1000, which is also the highest V_L_ for DLP2 of 0.92 (Figure 5).

#### 3.2.3. Biofilm Inhibition of DLP1 and DLP2

**Figure 6:**
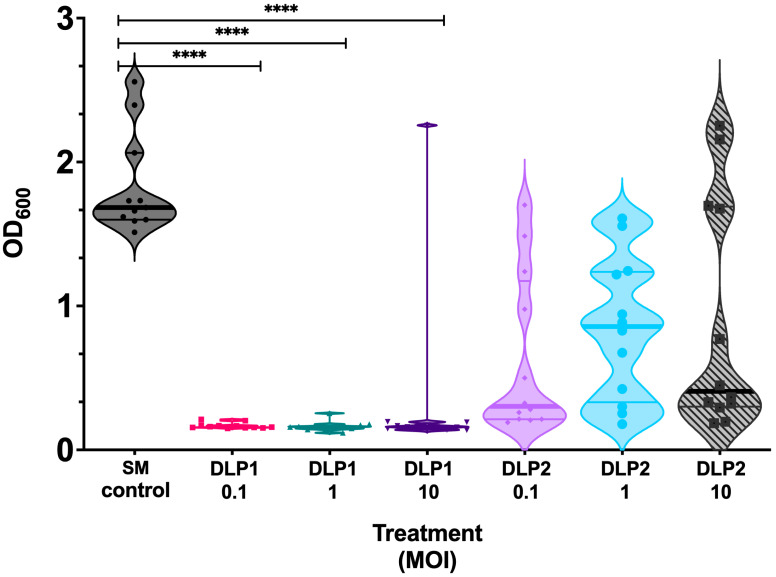
Biofilm inhibition of DLP1 and DLP2 at varying MOIs compared to the AB5075^cm^ control. Static biofilms were formed in a 96-well plate in the presence or absence of varying MOIs of DLP1 and DLP2 over 24 h and stained with 0.1% crystal violet. OD_600_ was measured. The data are presented as a violin plot showing the median and quartiles of a biological triplicate and technical quadruplicate experiment. Statistical significance is represented as ****, *p* < 0.0001.

Phages that prevent or reduce biofilm formation can be valuable in therapy, particularly for chronic infections with established biofilms. The examination of the biofilm prevention properties of each phage revealed that DLP1 and DLP2 have different biofilm suppression capabilities at each MOI tested (0.1, 1, 10) (Figure 6). DLP1 is overall more effective at preventing biofilm formation in AB5075^cm^, with a complete suppression of growth compared to the untreated control at all MOIs tested (*p* < 0.0001) (Figure 6). DLP2 does suppress AB5075^cm^ biofilm formation over a 24 h period as can be noted by the median of the violin plots in Figure 6, but the suppression fails to reach a statistical significance. Additionally, more overgrown wells were observed at all MOIs of DLP2 treatments, as shown with the spread of the violin plots compared to DLP1, thus suggesting that DLP2-mutant subpopulations are quickly overgrowing the wells (Figure 6).

#### 3.2.4. Stability of DLP1 and DLP2 across a pH and Temperature Range

**Figure 7:**
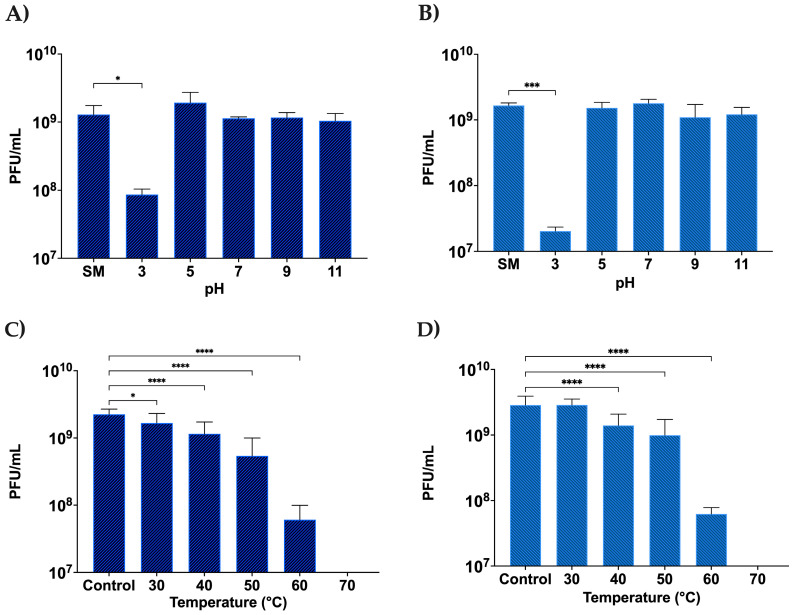
pH and temperature stability of DLP1 (**A**,**C**) and DLP2 (**B**,**D**) following an hour exposure. (**A**) pH stability of DLP1 compared to SM control (pH 7.4). (**B**) pH stability of DLP2 compared to SM control (pH 7.0). (**C**) DLP1 temperature stability compared to control (4 °C). (**D**) DLP2 temperature stability compared to control (4 °C). Temperatures 80 and 90 °C are not shown for simplicity. Error bars show the standard deviation of a biological and technical triplicate experiment. Statistical significance is represented as: *, *p* < 0.05; ***, *p* < 0.0005; ****, *p* < 0.0001.

The stability of phages under different pH levels and temperatures is important for phage therapy as the phages are not always guaranteed to be in a temperature-controlled environment, and depending on the delivery route, may require stability in low pH. Both DLP1 and DLP2 show good stability profiles across a range of pH levels, except for pH 3 which impacts both DLP1 and DLP2 (Figure 7). The viral titer of DLP1 was reduced by approximately one log (Figure 7A), where DLP2 titer decreased by nearly two logs following an hour of exposure to pH 3 (Figure 7B). This finding could account for the difficulty in obtaining micrographs of intact DLP2 when stained with 2% PTA. Both phages also showed moderate temperature stability until 60 °C was reached, at which point the titer dropped by approximately 1.5 logs for both DLP1 and DLP2 (Figure 7C,D). Both DLP1 and DLP2 are completely inactivated at 70 °C and above, with no phages recovered for temperatures 70, 80, and 90 °C (Figure 7C,D).

#### 3.2.5. Identification of DLP2 Receptor

An *A. baumannii* strain containing a deletion of the *wzc* gene to confer the loss of a capsule was mutagenized with MarTc and a pooled library of approximately 10,000 insertions was plated on LLB agar plates at a high density. A suspension of DLP1 or DLP2 phage was added to the lawn and surviving colonies were identified. There were no survivors to DLP1, but surviving colonies readily arose zones of DLP2 lysis. The MarTc insertion site in one survivor was mapped to the *carO* gene, encoding an outer membrane porin involved in uptake of ornithine and carbapenems [62,63]. The marTc insertion site in *carO* was at a position corresponding to amino acid 62 of the 247 amino acid protein. To verify the level of resistance, the titer of both DLP1 and DLP2 was determined on the mutant. The Δ*wzc, carO::MarTc* double mutant was completely resistant to DLP2, while DLP1 infection efficiency dropped by two logs compared to the AB5075 Δ*wzc* mutant (Table 2).

To independently verify the role of *carO*, a *carO::T26* insertion mutant from the ordered *A. baumannii* library was moved into the AB5075 Δ*wzc* mutant by natural transformation. This AB5075 Δ*wzc*, *carO::T26* mutant was sensitive to DLP1 with the same efficiency as the *carO::MarTc* double mutant, but highly resistant to DLP2 (Table 2). The AB5075 Δ*wzc*, *carO::T26* mutant was successfully complemented with *carO,* including its native ribosomal-binding site, using the pQF1266B plasmid. Complementation of the double mutant with *carO* restored DLP1 and DLP2 infectivity to near wild-type levels, confirming that the receptor for DLP2 is indeed CarO (Table 2).

### 3.3. Genomic Analysis

#### 3.3.1. Restriction Fragment Length Polymorphism Analysis (RFLP)

**Figure 8:**
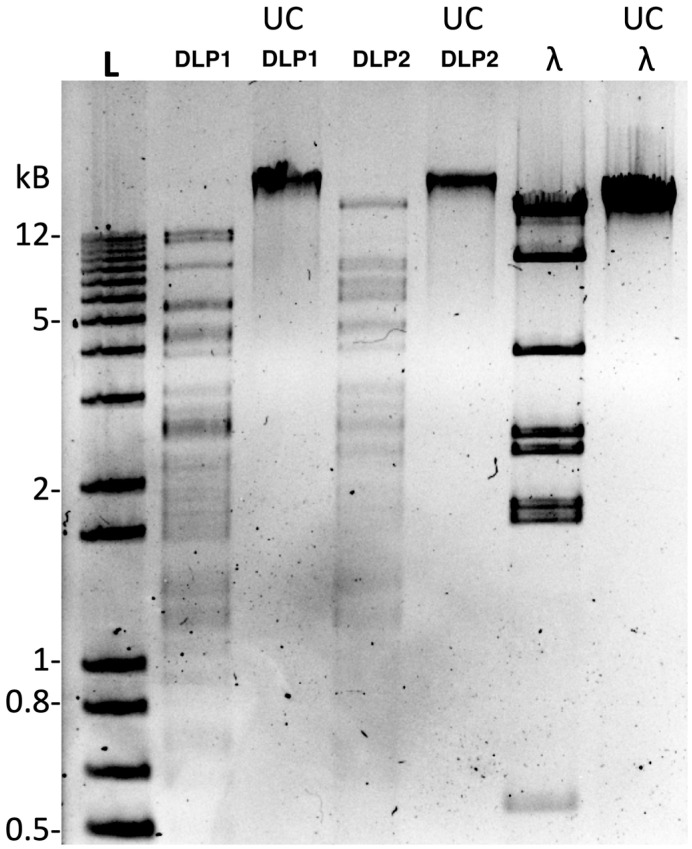
RFLP digestion of DLP1, DLP2, and λ control with *Nde*I. A total of 1 μg of phage gDNA was digested for 5 h at 37 °C with *Nde*I and separated on a 1.2% agarose gel in 1x TAE (pH 8.0). L: 1 kb Plus GeneRuler DNA Ladder (Thermo Scientific); UC: uncut control.

**Figure 9:**
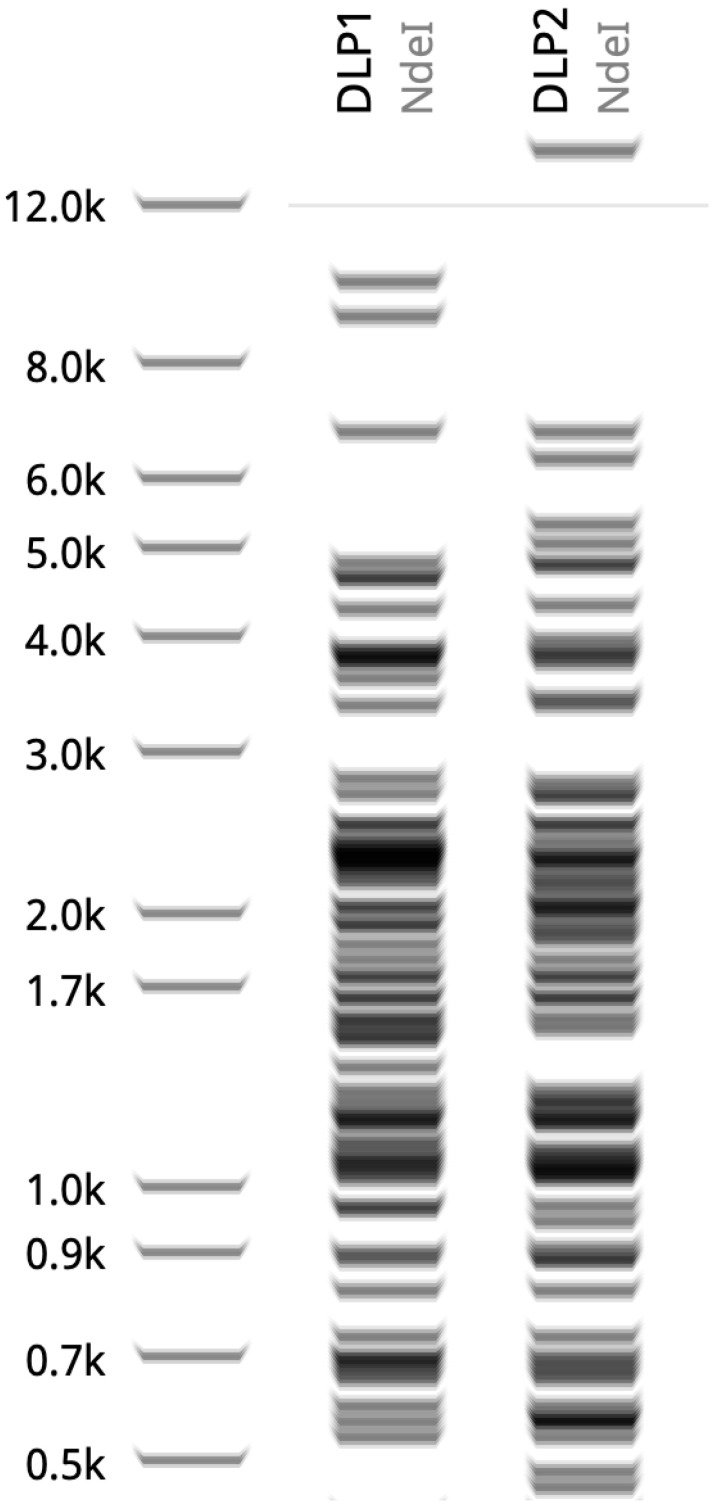
In silico digestion of DLP1, and DLP2 with *Nde*I using Geneious Prime. Ladder is 1 kb Plus GeneRuler DNA Ladder.

RFLP analysis using a panel of 16 restriction enzymes shows complete resistance to all enzymes except *Nde*I, thus suggesting similar DNA modifications in both phages (Figure 8). This finding contrasts the only RFLP data reported for the T4-like *A. baumannii* phage ZZ1, which can be digested by *Hind*III, *Eco*RI, and *Eco*RV [64]. The digestion of DLP1 and DLP2 gDNA over 5 h with *Nde*I shows banding differences between 10–20 kb and 3–4 kb (Figure 8). This digestion pattern is similar to the in silico prediction of DLP1 and DLP2 gDNA digested with NdeI (Figure 9), though the banding pattern is slightly shifted due to the inability to change the agarose percent with the prediction. There is a double band evident in the DLP1 actual and in silico NdeI digest (~9 and 10 kb), which is missing in the DLP2 digest (Figure 8 and Figure 9). DLP1 also has a ~7 kb fragment which is present in both simulated and actual digests (Figure 8 and Figure 9). There is a high molecular weight band (~16 kb) present in the DLP2 actual and in silico digest, as well as a doublet around 6 and 6.5 kb (Figure 8 and Figure 9).

The enzyme activity was greatly reduced compared to the λ gDNA control, which is digested within 30 min compared to 5 h with DLP1 and DLP2 (data not shown). This result suggests a DNA modification of DLP1 and DLP2 DNA that is causing steric hindrance to the enzyme, slowing its ability to digest the DNA.

#### 3.3.2. Genome Features of DLP1 and DLP2

The phages were sequenced and trimmed, and the filtered reads were assembled into 164,355 and 165,122 bp contigs for DLP1 and DLP2, respectively. The alignment of 1,431,454 trimmed, merged paired reads back to the DLP1 contig resulted in 99.3% of the reads used and 99.9% pairwise ID with a mean coverage of 1165x. The alignment of DLP2 trimmed, merged paired reads (1,646,288) back to the DLP2 contig resulted in 88.7% of the reads used with 99.9% pairwise ID and 1620x coverage. The DNA packaging style of the phages was determined using the program PhageTerm analysis and the trimmed and filtered DLP1 and DLP2 paired reads against each genome. The results show that both phages are circularly permuted and terminally redundant, a common genomic characteristic of T4-like phages [65].

**Figure 10:**
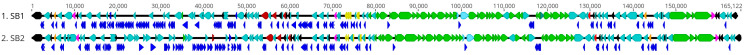
Genome maps of DLP1 and DLP2. Functional assignment of the predicted proteins encoded by each open reading frame is as follows: hypothetical (navy), morphogenesis (green), transcription and translation (teal), regulatory (black), lysis (pink), terminase (light blue), recombination (dark red), resistance (orange), tRNA (yellow), and virulence (red).

The DLP1 genome has a 36.8% GC content, encodes 248 proteins, and has a coding density of 94.3% (Figure 10; Supplementary Table S1). In contrast, the DLP2 genome has a GC content of 36.7%, encoding 248 proteins and a coding density of 94.1% (Figure 10; Supplementary Table S2). Identification of tRNAs with tRNAScan-SE revealed DLP1 encodes ten tRNAs and DLP2 encodes eight. Both phages encode the same set of seven tRNAs: Sup (CTA), Cys (GCA), Leu (TAG), Arg (TCT), Ser (TGA), Phe (GAA), Trp (CCA). Phage DLP1 encodes an additional Ser (GCT), and an Ile2 (CAT); whereas DLP2 encodes an Asn (GTT) tRNA.

Both DLP1 and DLP2 encode a very similar set of proteins, including many hypotheticals (123 for DLP1 and 117 for DLP2) which cannot be assigned functions at this time (Figure 10, dark blue; Supplementary Tables S1 and S2). Although there have been significant advances in high-throughput sequencing, there is still a limited amount of functional information on viral proteins [66]. Often, genes encoding hypothetical proteins dominate a novel phage assembly, as we show with the annotation of the DLP1 and DLP2 genomes. Analysis of the genomes suggest that these are strictly lytic phages as no repressors of the lysogenic cycle were identified. All the proteins involved in transcription and translation, virion morphogenesis, and regulation for DLP1 and DLP2 were closely related to other T4-like *Acinetobacter* phages in the NCBI database, such as Stupor (accession MN662249.1), AB-Navy1 (accession OL770258.1), and AB-Navy97 (accession OL770261.1) (Figure 10, dark blue; Supplementary Tables S1 and S2). A further comparison to the T4-like *Acinetobacter* phages is presented in Section 3.3.4.

Surprisingly, both DLP1 and DLP2 encode two putative resistance proteins: dihydrofolate reductase (DHFR) and the multidrug resistance transporter EmrE (efflux-multidrug resistance E). Both resistance proteins encoded by each phage were identified with InterProScan using the abovementioned databases, and further analyzed with PHYRE2 and EBI Protein Similarity Search.

The DHFR protein encoded by the phages corresponds to gp227 (ADLP1_227) and gp226 (ADLP2_226) in DLP1 and DLP2, respectively. The DHFR proteins encoded by the phages share 99.5% AA identity when aligned with CLUSTAL and have identical database hits with InterProScan (Table 3; Supplementary Tables S1 and S2). The top hits with PHYRE2 analysis on the DLP1 and DLP2 DHFR proteins are to the template d1juva_ (fold: Dihydrofolate reductase-like) with 100% confidence, and 46 and 45 % ID across the template, respectively (Supplementary Figures S1 and S2). All remaining hits against these proteins with PHYRE2 are to DHFR proteins with very high confidence. Furthermore, the EBI results also support this prediction, with the top DLP1 and DLP2 hit from the PDB (1juv) to the DHFR protein of *E. coli* T4 with an E-value of 5.3 × 10^−11^ and 3.1 × 10^−10^, respectively (Supplementary Figures S3 and S4).

The encoded EmrE protein corresponds to gp8 (ADLP1_008 and ADLP2_008) for both phages which share 96.5 % ID across the proteins. Each putative EmrE protein had seven database hits with InterProScan (Table 4), all regarding multidrug transporters or transmembrane regions. PHYRE2 analysis on the predicted EmrE proteins hit to the same template (d1s7ba_) of the multidrug efflux transporter EmrE with 100% confidence and 32 and 31% ID for DLP1 and DLP2, respectively (Supplementary Figures S5 and S6). Further analysis on the proteins with the EBI search revealed the DLP1 EmrE top hit is to a quaternary ammonium transporter (A0A6C0Y7S0; AFDB) from *Acinetobacter indicus* (gene: FSC09_15345) with an E-value of 2.9 × 10^−19^ (Supplementary Figure S7). For DLP2, EmrE top hit is also to a quaternary ammonium transporter (A0A6N0LIZ1; AFDB) from *Acinetobacter* sp. 10FS3-1 (gene: E5Y90_15705) with an E-value of 3.3 × 10^−19^ (Supplementary Figure S8).

Both phages encode the putative virulence factor YbiA (ADLP1_205 and ADLP2_205), which has been shown to cause defects in *E. coli* swarming when mutated [67]. The DLP1 and DLP2 proteins share 100% identity and are supported by YbiA protein domain hits in the following databases: SUPERFAMILY (YbiA-like, IPR037238), CDD (NADAR, IPR012816), and Gene3D (YbiA-like_sf, IPR037238).

#### 3.3.3. Comparison of DLP1 and DLP2 Genomes and Tail Fibers

**Figure 11:**
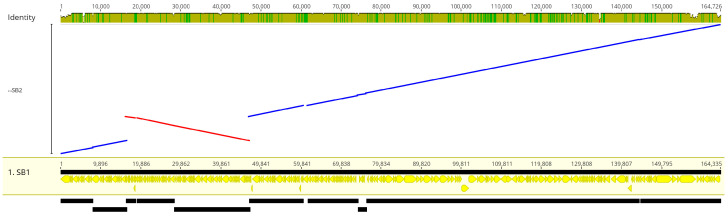
LASTZ alignment of the DLP2 forward (blue) and reverse (red) genome against DLP1. The DLP2 alignment shows a 31 kb inversion compared to DLP1. The inverted section of DLP2 encodes genome replication and modification genes.

To determine the average nucleotide identity (ANI) shared between DLP1 and DLP2, the ANI calculator was used. An ANI analysis of the genomes shows high nucleotide identity between DLP1 and DLP2, with a mean identity (ID) of 97.85% and median of 98.34% (Supplementary Figure S9). A genomic comparison of DLP1 and DLP2 with a LASTZ alignment reveals identity conservation across the entire genome, as found with the ANI analysis, except there is a 31 kb inversion of the replication and DNA modification module of DLP2 compared to DLP1 (Figure 11).

The short and long tail fibers of DLP1 and DLP2 were compared to analyze what is potentially contributing to differences in host range and the presence of a halo with the plaque formation of DLP1, which is typically associated with a pectate lyase domain. Clustal Omega analysis of the short tail fibers of DLP1 (gp167) and DLP2 (gp167) shows a 96.7% amino acid (AA) identity (Supplementary Figure S10). There are 16 AA differences between the DLP1 and DLP2 short tail fibers, with eight changes found in the receptor-binding domain of DLP1 compared to DLP2: F316Y, P429S, N433T, R435Q, F451W, K456R, P486S, and T492S. These amino acid changes could alter protein folding at the receptor-binding domain and account for the host range differences noted in the characterization of these phages.

Three AA differences between DLP1 (gp158) and DLP2 (gp158) long tail fibers were identified in the Clustal Omega protein comparison (Supplementary Figure S11). A single AA change was found in the T4 lysozyme domain of DLP2 compared to DLP1 (I198V), which may alter function due to size differences between the amino acids, but both have aliphatic side chains. The other two AA changes were found at AA 350 and 351, which are outside of the functional domains of the proteins. Compared to DLP1, the DLP2 AA changes are S350V and A351V. The first AA change between DLP1 and DLP2 may have an impact in protein folding due to the sidechain properties of serine (polar uncharged) compared to valine (hydrophobic).

#### 3.3.4. Genomic Analysis of DLP1 and DLP2 against Published Acinetobacter Phages

**Figure 12:**
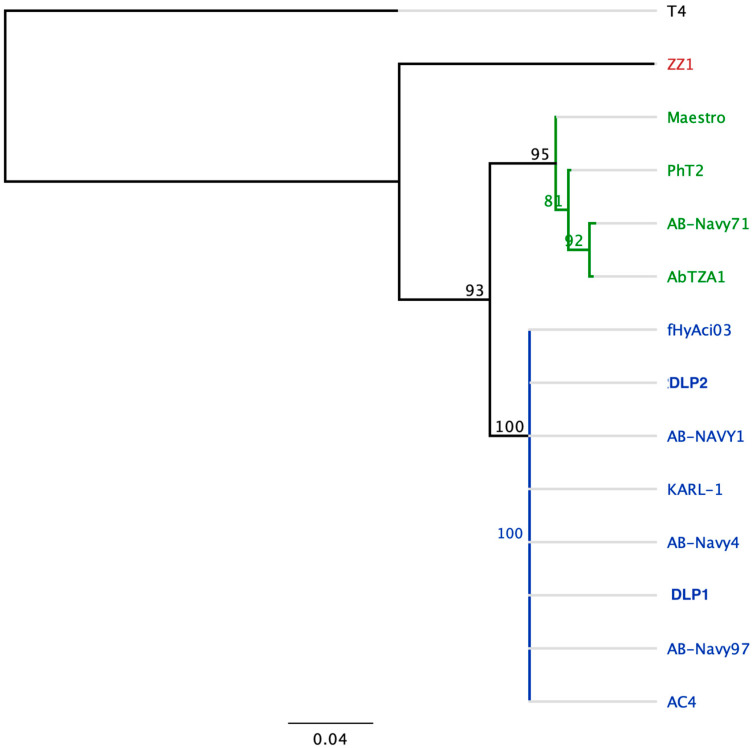
A rooted tree of the large terminase proteins of 11 published *Acinetobacter* T4-like phages. PHYML was used on a Clustal Omega alignment of the TerL protein, using *Escherichia* phage T4 to root the tree. Branch support is shown from 100 bootstrap replicates. Blue represents the *Lazarusvirus* clade, green represents the *Hadassahvirus* clade. Phage ZZ1 (red) forms the *Zedzedvirus* clade.

Both phages have T4 TerL hits from the HAMAP database, suggesting that both phages belong to the *Straboviridae* family. Further classification of the DLP1 and DLP2 phages was accomplished using the large terminase protein due to its conservation across dsDNA tailed phages. A Clustal Omega protein alignment and PHYLM tree of the large terminase of DLP1 (gp174) and DLP2 (gp174) against a panel of characterized *Acinetobacter* T4-like phages were conducted using the *Escherichia* phage T4 to root the tree (Figure 12, Table 5). The *Acinetobacter* T4-like phages are very closely related to each other, forming three distinct groups (Figure 12). Both DLP1 and DLP2 group together with phages fHyAci03, AB-Navy4, AB-Navy-97, AB-Navy-1, AC4, and KARL-1 which belong to the family *Straboviridae,* subfamily *Twarogvirinae* genus *Lazarusvirus* (Figure 12; blue). The second clade formed with phages Maestro, AB-Navy-71, AbTZA1, and PhT2 which forms the *Hadassahvirus* genus (Figure 12; green). *Acinetobacter* phage ZZ1 formed its own clade, representing the *Zedzedvirus* genus, thus suggesting divergence from the *Lazarusvirus* and *Hadassahvirus* phages (Figure 12; red).

**Figure 13:**
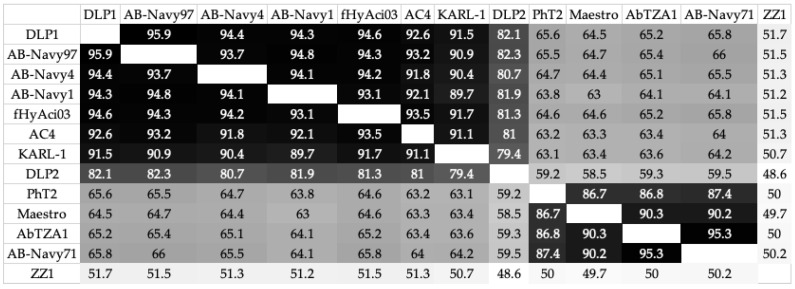
A heat map (% ID) of a MAFFT whole genome alignment using the *Acinetobacter* T4-like phages.

**Figure 14:**
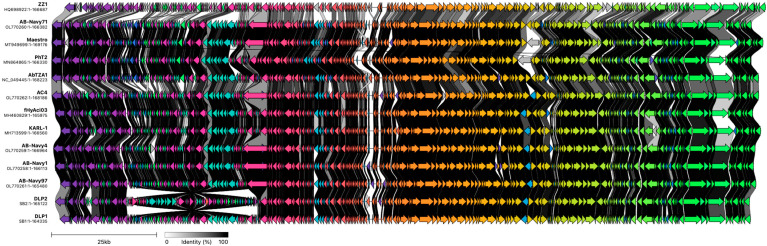
A Clinker gene cluster comparison of *Acinetobacter* T4-like phages. Comparison of whole genomes for *Acinetobacter* phages DLP1 and DLP2 against 11 other T4-like phages with percent amino acid identity represented by greyscale links between genomes. Each similarity group is assigned a unique color.

Further comparison of the DLP1 and DLP2 genomes with MAFFT against the T4-like *Acinetobacter* phages shows a high degree of identity across the entirety of the genomes, with ZZ1 showing the greatest breakdown in % identity compared to the other phages (Figure 13). Due to the ~31 kb inversion of DLP2, the MAFFT % identity heatmap shows DLP2 has a lower % identity against the *Lazarusvirus* grouping (79.4–82.4% ID) compared to DLP1 (82.4–95.9% ID) (Figure 13). Visualization of the phage genome alignment with Clinker shows a typical modular conservation of genes with all phages, but % identity decreases between the clades, as represented with a lighter grey band between the genes of each genome (Figure 14). The ~31 kb inversion of DLP2 is clearly evident when compared to the other T4-like phages (Figure 14).

## 4. Discussion

The characterization of *A. baumannii* bacteriophages is on the rise due to the increased popularity of this treatment modality. There have been a number of T4-like *A. baumannii* phages described recently (Table 5), with some being involved in the actual treatment of an *A. baumannii* infection in a human [17]. The host ranges of both DLP1 and DLP2 are modest at 15 and 20%, but similar to other previously characterized T4-like *Acinetobacter* phages that have host range data (e.g., KARL-1, 40%; ZZ1, 13%; fHyAci03, 16%; and PhT2, 28%) [64,68,69,71]. The host ranges of DLP1 and DLP2 can be further expanded using The Appelmans Protocol, which passages a cocktail of phages on a single strain of bacteria to isolate recombinant phages with expanded host ranges [11]. Additionally, the EOP of DLP2 on different strains can be increased through the serial passage of the phage on that host. Coevolutionary phage training has been shown to enable greater bacterial suppression and delay evolution of phage resistance in vitro [72]. The use of these methods on DLP2 could help enhance its efficiency against different *A. baumannii* strains.

*A. baumannii* is an encapsulated bacterium which can undergo phase variation, switching from an avirulent transparent (AV-T) to a virulent opaque (VIR-O) phase [31,73]. The VIR-O phase exhibits a thicker capsule layer and is shown to be more virulent in *Galleria mellonella* and mouse lung infection models [19,31,73]. Additionally, mutants that lack capsules can be frequently isolated from stationary *A. baumannii* overnight cultures. Phase variation and capsule mutants can complicate phage infection studies due to the varying capsule thickness which can impact phage infection. The thick VIR-O capsule can prevent certain phages, such as DLP1 and DLP2, from reaching their receptor, but it can also be required for successful infection by other phages [74]. A capsule mutant was used for phage hunting to enable the isolation of more phages for engineering. Although DLP1 and DLP2 tend to infect capsule mutants, with the exception of ATCC 19606, it has been shown to be beneficial to isolate phages targeting both the VIR-O phase and capsule mutants to ensure sufficient coverage for these mutants during treatment. This approach was successfully used in the treatment of a 68-year-old diabetic man with a disseminated *A. baumannii* infection, where phages that target capsule mutants were included in the cocktail [12,17,75].

The burst size of phages DLP1 (235 PFU/cell) and DLP2 (26 PFU/cell) are very different. Only two T4-like *A. baumannii* phages, ZZ1 and KARL-1, have burst size information available, which reveals burst sizes of 200 and 39 PFU/cell, respectively [64,69]. It should be noted that the comparison of burst sizes between different phages can be challenging due to the different methodologies, conditions, and equipment used by each research laboratory. Other types of *A. baumannii* phages with large burst sizes have been documented, for example: AS12; 300 [76]; TAC1, 454 [34]; vB_AbaM_IME285, 450 [77]; AB1, 409 [78]; Abp1, 350 [79]. The burst sizes of DLP1 and DLP2 are not extraordinary, but the large burst of DLP1 in particular suggests that it could be a promising candidate for therapeutic use.

The swift rise in mutant outgrowths observed with DLP2 compared to DLP1 in the biofilm-inhibition experiment (Figure 6) suggested that DLP1 and DLP2 use different host receptors. This was confirmed using a transposon mutant library of AB5075 Δ*wzc,* which identified CarO as the DLP2 receptor. No receptor mutants were identified for DLP1, but it is interesting to note that DLP1 infection efficiency decreased on the Δ*wzc, carO* mutant compared to the Δ*wzc* mutant control. This suggests that DLP1 may be able to use CarO as a secondary receptor. The use of phages with different receptors is ideal in a cocktail as they provide protection against phage-resistant mutants [80]. The ability of DLP1 to effectively suppress biofilm formation and the planktonic growth of AB5075^cm^ makes this phage particularly interesting for use in therapy. However, an additional phage targeting the VIR-O phase of AB5075 would be required to form a robust cocktail against *A. baumannii* [12,80].

There are two potential antibiotic resistance proteins encoded by DLP1 and DLP2: DHFR and EmrE, as reported in Section 3.3.2 of the results. The presence of DHFR encoded by both DLP1 and DLP2 was initially surprising as it has been shown to be used by some temperate phages to increase the trimethoprim resistance of the lysogen [81]. The function of DHFR is to catalyze the NADPH-dependent reduction of dihydrofolate to tetrahydrofolate (IPR012259). This is an essential step in the de novo synthesis of glycine, purines, and deoxythymidine phosphate, all of which are precursors required for DNA synthesis [82]. The classical *E. coli* phage T4 encodes DHFR, which has been identified to be involved in thymidylate metabolism and as a protein component of the tail baseplate [83,84]. Thus, DLP1 and DLP2 likely use the encoded DHFR for nucleotide recycling during replication and as a structural protein.

It is puzzling what the putative multidrug resistance transporter EmrE would be used for by the phages; however, this protein is encoded by all *Acinetobacter* T4-like phages, including those used in the treatment of a human infection [75]. Although the phages may not be using these encoded proteins for antimicrobial resistance, lysis of the host cell will result in the release of these genes into the environment where they can be taken up by sensitive bacteria with natural transformation and expressed as a resistance factor.

Further expanding on this, both phages encode YbiA, which has been shown to cause defects in E. coli swarming when mutated [67]. A Stenotrophomonas temperate phage was found to encode YbiA which was shown to partially restore swarming motility in a ybiA^−^ *E. coli* [81]. The presence of this gene and the putative resistance genes highlights the importance of characterizing the phages prior to use. Luckily, these phages are lytic, so lysogenic conversion of their host is not applicable.

Comparison of the DLP1 and DLP2 genomes to 11 other *Acinetobacter* T4-like phages predictably reveals a high conservation of identity between all functional gene modules (Figure 14). Analyzing and comparing the genomes of this group of phage raises interesting questions around gene conservation and the amount of recombination, or lack of, that occurs in each geographic location from which the phages were isolated from.

Further investigation into the DNA modification of DLP1 and DLP2 is needed to determine the exact DNA modifications these phages employ for restriction enzyme resistance. In *E. coli* phage T4, glucosyl-hydroxymethylcytosine completely replaces cytosine in the DNA and protects phage DNA from cleavage by restriction enzymes upon infection of the host cell [85]. To date, only *A. baumannii* phage ZZ1 has RFLP data presented, which shows that the genome is sensitive to *Hind*III, *Eco*RI, and *Eco*RV [64]. The restriction panel used against ZZ1 gDNA was not documented so it is unknown if it is susceptible to *Nde*I.

## 5. Conclusions

The T4-like *A. baumannii* phages DLP1 and DLP2 show good therapeutic potential for inclusion in a phage cocktail. Both candidates have significant burst sizes compared to other *Acinetobacter* T4-like phages characterized to date and display promising infection dynamics. Both phages exhibit modest host ranges like other T4-like *Acinetobacter* phages, which can be expanded using specific techniques to further enhance the therapeutic potential of these phages. Together, DLP1 and DLP2 show promise for use as therapeutic phages against antimicrobial-resistant *A. baumannii* infections.

## Figures and Tables

**Table 2:** Infection characteristics of DLP1 and DLP2 against carO mutants.

Strain	DLP1 (PFU/mL)	DLP2 (PFU/mL)
AB5075 Δ*wzc*	2.9 × 10^8^	7.4 × 10^8^
AB5075 Δ*wzc, carO::MarTc*	1.2 × 10^6^	0
AB5075 Δ*wzc*, *carO::T26*	1.0 × 10^6^	0
AB5075 Δ*wzc*, *carO::T26*/pQF1266B	2.4 × 10^6^	0
AB5075 Δ*wzc*, *carO::T26*/pQF1266B + *carO*	1.9 × 10^8^	2.0 × 10^7^

**Table 3:** InterProScan database hits on DLP1 and DLP2 DHFR. The data represent findings for both phages due to identical search results.

Database	InterPro ID	InterPro Name
CDD	IPR001796	DHFR_dom
Gene3D	IPR024072	DHFR-like_dom_sf
Panther	IPR012259	DHFR
Pfam	IPR001796	DHFR_dom
PRINTS	PR00070	DHFR
PROSITE_PROFILES	IPR001796	DHFR_dom
Superfamily	IPR024072	DHFR-like_dom_sf

**Table 4:** InterProScan database hits on DLP1 and DLP2 EmrE. The data represent findings for both phages due to duplicate search results.

Database	Hit Description
Gene3D	G3DSA:1.10.3730.20
Panther	SMR family proton-dependent drug efflux transporter SUGE
Pfam	Multi_Drug_Res
Superfamily	Multidrug resistance efflux transporter EmrE
TMHMM	unknown
PHOBIUS	Transmembrane, cytoplasmic and non-cytoplasmic domains
SIGNALP_EUK	SignalP-TM

**Table 5:** Comparison of T4-like *A. baumannii* phages published to date.

Phage	Genome (bp)	ORFs	tRNAs	GC (%)	Accession	Reference
DLP1	164,335	248	10	36.8	OP946501	This study
DLP2	165,122	248	8	36.7	OP946502	This study
fHyAci03	165,975	247	8	36.8	MH460829	[68]
KARL-1	166,560	253	7	36.8	MH713599	[69]
ZZ1	166,687	256	8	34.4	HQ698922	[64,70]
PhT2	166,330	255	9	36.4	MN864865	[71]
Maestro ^1^	169,176	264	7	36.3	MT949699	[12,17]
AC4 ^1^	168,186	250	9	36.7	OL770262	[12,17]
AB-Navy1 ^1^	166,113	247	8	36.7	OL770258	[12,17]
AB-Navy4 ^1^	166,964	246	8	36.8	OL770259	[12,17]
AB-Navy71 ^1^	166,382	252	8	36.4	OL770260	[12,17]
AB-Navy97 ^1^	165,480	244	9	36.7	OL770261	[12,17]
ABTZA1	168,223	253	6	36.3	MK278860	[14]

^1^ Used in a phage cocktail in the successful treatment of a disseminated *A. baumannii* infection.

## Data Availability

Genomic sequences are available with the following accession numbers: OP946501(DLP1) and OP946502 (DLP2).

## Supplementary Materials

The following supporting information can be downloaded at: https://www.mdpi.com/article/10.3390/v15030739/s1, Figure S1: PHYRE2 analysis of DLP1 DHFR; Figure S2: PHYRE2 analysis of DLP2 DHFR; Figure S3: EBI Protein Similarity Search hits of DLP1 DHFR; Figure S4: EBI Protein Similarity Search hits of DLP2 DHFR; Figure S5: PHYRE2 analysis of DLP1 EmrE; Figure S6: PHYRE2 analysis of DLP2 EmrE; Figure S7: EBI Protein Similarity Search hits of DLP1 EmrE; Figure S8: EBI Protein Similarity Search hits of DLP2 EmrE; Figure S9: Average Nucleotide Identity comparison of DLP1 and DLP2; Figure S10: Clustal Omega alignment of DLP1 (gp167) and DLP2 (gp167) short tail fiber protein sequences; Figure S11: Clustal Omega alignment of DLP1 (gp158) and DLP2 (gp158) long tail fiber proteins; Table S1: DLP1 annotation table; Table S2: DLP2 annotation table.
