# Supplementary material for: Characterization of Virulent T4-Like Acinetobacter baumannii Bacteriophages DLP1 and DLP2
Source: Viruses. 2023 Mar 13;15(3):739. doi: 10.3390/v15030739 (PMC10051250; doi:10.3390/v15030739)
Supplement: Supplementary file 1 [file viruses-15-00739-s001.zip › Supplementary files/SFigure 6_PHYRE2 DLP2 gp008 hit_report.pdf]

Phyre^2

Email	dlpeters@ualberta.ca
Description	SB2_-_putative_quaternary_ammonium_compound-resistance_protein_translation_Acinetobacter_phage_SB2,_complete_genome
Date	Fri Jan 6 04:30:02 GMT 2023
Unique Job ID	2752e32602c05862

Detailed template information

#	Template	Alignment Coverage	3D Model	Confidence	% i.d.	Template Information
1	d1s7ba_	Alignment	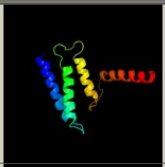	100.0	31	**Fold:** Multidrug resistance efflux transporter EmrE<br>**Superfamily:** Multidrug resistance efflux transporter EmrE<br>**Family:** Multidrug resistance efflux transporter EmrE<br>**PDB entry:** PDBe RCSB PDBj
2	c2i68B_	Alignment	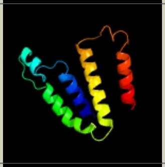	99.9	32	**PDB header:**transport protein<br>**Chain:** B: **PDB Molecule:**protein emre;<br>**PDBTitle:** cryo-em based theoretical model structure of transmembrane2 domain of the multidrug-resistance antiporter from e. coli3 emre<br>**PDB Entry:** PDBe RCSB PDBj
3	c6wk9B_	Alignment	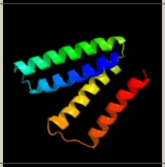	100.0	22	**PDB header:**transport protein<br>**Chain:** B: **PDB Molecule:**multidrug resistance protein, smr family;<br>**PDBTitle:** crystal structure of gdx-clo from small multidrug resistance family of2 transporters in complex with octylguanidinium<br>**PDB Entry:** PDBe RCSB PDBj
4	c7pafA_	Alignment	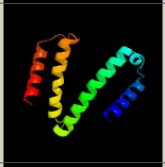	97.7	18	**PDB header:**transport protein<br>**Chain:** A: **PDB Molecule:**licb protein;<br>**PDBTitle:** streptococcus pneumoniae choline importer licb in lipid nanodiscs<br>**PDB Entry:** PDBe RCSB PDBj
5	c5y79A_	Alignment	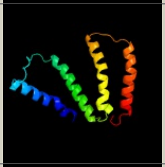	97.9	14	**PDB header:**transport protein<br>**Chain:** A: **PDB Molecule:**putative hexose phosphate translocator;<br>**PDBTitle:** crystal structure of the triose-phosphate/phosphate translocator in2 complex with 3-phosphoglycerate<br>**PDB Entry:** PDBe RCSB PDBj
6	c5ogeE_	Alignment	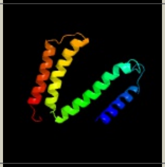	97.7	13	**PDB header:**membrane protein<br>**Chain:** E: **PDB Molecule:**gdp-mannose transporter 1;<br>**PDBTitle:** crystal structure of a nucleotide sugar transporter<br>**PDB Entry:** PDBe RCSB PDBj
7	c5i20E_	Alignment	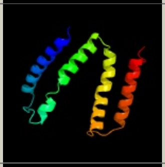	98.1	13	**PDB header:**membrane protein<br>**Chain:** E: **PDB Molecule:**uncharacterized protein;<br>**PDBTitle:** crystal structure of protein<br>**PDB Entry:** PDBe RCSB PDBj
8	c6ukjA_	Alignment	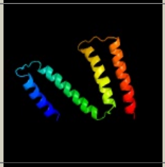	97.7	13	**PDB header:**membrane protein<br>**Chain:** A: **PDB Molecule:**chloroquine resistance transporter;<br>**PDBTitle:** single-particle cryo-em structure of plasmodium falciparum chloroquine2 resistance transporter (pfcrt) 7g8 isoform<br>**PDB Entry:** PDBe RCSB PDBj
9	c5i20C_	Alignment	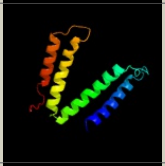	97.7	10	**PDB header:**membrane protein<br>**Chain:** C: **PDB Molecule:**uncharacterized protein;<br>**PDBTitle:** crystal structure of protein<br>**PDB Entry:** PDBe RCSB PDBj
10	c6oh2A_	Alignment	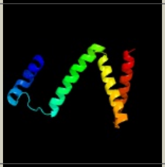	98.1	8	**PDB header:**transport protein<br>**Chain:** A: **PDB Molecule:**cmp-sialic acid transporter;<br>**PDBTitle:** x-ray crystal structure of the mouse cmp-sialic acid transporter in2 complex with cmp, by lipidic cubic phase<br>**PDB Entry:** PDBe RCSB PDBj
11	c6i1rA_	Alignment	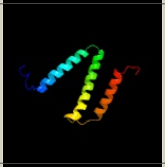	97.9	9	**PDB header:**membrane protein<br>**Chain:** A: **PDB Molecule:**cmp-sialic acid transporter 1;<br>**PDBTitle:** crystal structure of cmp bound cst in an outward facing conformation<br>**PDB Entry:** PDBe RCSB PDBj
						**PDB header:**membrane protein

12	c4oo9A_	Alignment	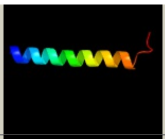	20.6	26	**Chain:** A: **PDB Molecule:**metabotropic glutamate receptor 5, lysozyme, metabotropic<br>**PDBTitle:** structure of the human class c gpcr metabotropic glutamate receptor 52 transmembrane domain in complex with the negative allosteric3 modulator mavoglurant<br>**PDB Entry:** PDBe RCSB PDBj
13	c6rfsX_	Alignment	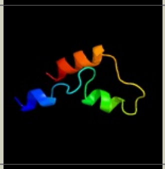	16.2	21	**PDB header:**oxidoreductase<br>**Chain:** X: **PDB Molecule:**subunit nuxm of nadh:ubiquinone oxidoreductase (complex i);<br>**PDBTitle:** cryo-em structure of a respiratory complex i mutant lacking ndufs4<br>**PDB Entry:** PDBe RCSB PDBj
14	c3mp7B_	Alignment	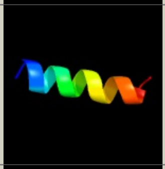	12.1	29	**PDB header:**protein transport<br>**Chain:** B: **PDB Molecule:**preprotein translocase subunit sece;<br>**PDBTitle:** lateral opening of a translocon upon entry of protein suggests the2 mechanism of insertion into membranes<br>**PDB Entry:** PDBe RCSB PDBj
15	c6yp7J_	Alignment	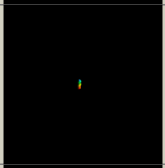	10.9	7	**PDB header:**photosynthesis<br>**Chain:** J: **PDB Molecule:**photosystem ii reaction center protein j;<br>**PDBTitle:** psii-lhcii c2s2 supercomplex from pisum sativum grown in high light2 conditions<br>**PDB Entry:** PDBe RCSB PDBj
16	c6yp7j_	Alignment	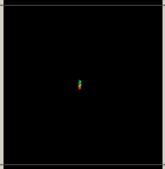	10.9	7	**PDB header:**photosynthesis<br>**Chain:** J: **PDB Molecule:**photosystem ii reaction center protein j;<br>**PDBTitle:** psii-lhcii c2s2 supercomplex from pisum sativum grown in high light2 conditions<br>**PDB Entry:** PDBe RCSB PDBj
17	c5xnmj_	Alignment	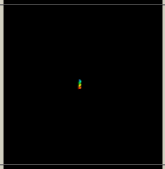	10.7	7	**PDB header:**membrane protein<br>**Chain:** J: **PDB Molecule:**photosystem ii reaction center protein j;<br>**PDBTitle:** structure of unstacked c2s2m2-type psii-lhcii supercomplex from pisum2 sativum<br>**PDB Entry:** PDBe RCSB PDBj
18	c7shfB_	Alignment	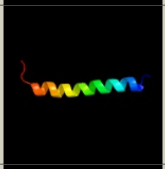	10.5	3	**PDB header:**signaling protein<br>**Chain:** B: **PDB Molecule:**g-protein coupled receptor 158;<br>**PDBTitle:** cryo-em structure of gpr158 coupled to the rgs7-gbeta5 complex<br>**PDB Entry:** PDBe RCSB PDBj
19	c6l85A_	Alignment	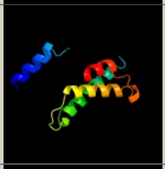	9.6	6	**PDB header:**membrane protein<br>**Chain:** A: **PDB Molecule:**phosphate transporter;<br>**PDBTitle:** the sodium-dependent phosphate transporter<br>**PDB Entry:** PDBe RCSB PDBj
20	c3jcuj_	Alignment	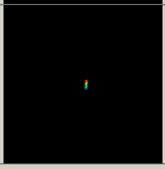	8.9	7	**PDB header:**membrane protein<br>**Chain:** J: **PDB Molecule:**photosystem ii reaction center protein j;<br>**PDBTitle:** cryo-em structure of spinach psii-lhcii supercomplex at 3.2 angstrom2 resolution<br>**PDB Entry:** PDBe RCSB PDBj
21	c7dtvA_	Alignment	not modelled	8.7	20	**PDB header:**membrane protein<br>**Chain:** A: **PDB Molecule:**extracellular calcium-sensing receptor;<br>**PDBTitle:** human calcium-sensing receptor bound with l-trp and calcium ions<br>**PDB Entry:** PDBe RCSB PDBj
22	c7vcfK_	Alignment	not modelled	8.6	18	**PDB header:**translocase<br>**Chain:** K: **PDB Molecule:**toc10;<br>**PDBTitle:** cryo-em structure of chlamydomonas toc-tic supercomplex<br>**PDB Entry:** PDBe RCSB PDBj
23	c6kacJ_	Alignment	not modelled	7.9	24	**PDB header:**photosynthesis<br>**Chain:** J: **PDB Molecule:**photosystem ii reaction center protein j;<br>**PDBTitle:** cryo-em structure of the c2s2-type psii-lhcii supercomplex from2 chlamydomonas reihardtii<br>**PDB Entry:** PDBe RCSB PDBj
24	c7pi0J_	Alignment	not modelled	6.8	24	**PDB header:**photosynthesis<br>**Chain:** J: **PDB Molecule:**photosystem ii reaction center protein j;<br>**PDBTitle:** unstacked compact dunaliella psii<br>**PDB Entry:** PDBe RCSB PDBj
25	c7pi0j_	Alignment	not modelled	6.8	24	**PDB header:**photosynthesis<br>**Chain:** J: **PDB Molecule:**photosystem ii reaction center protein j;<br>**PDBTitle:** unstacked compact dunaliella psii<br>**PDB Entry:** PDBe RCSB PDBj
26	c5doqC_	Alignment	not modelled	6.6	17	**PDB header:**oxidoreductase<br>**Chain:** C: **PDB Molecule:**putative membrane protein;<br>**PDBTitle:** the structure of bd oxidase from geobacillus thermodenitrificans<br>**PDB Entry:** PDBe RCSB PDBj
27	c5ir6C_	Alignment	not modelled	6.6	17	**PDB header:**oxidoreductase<br>**Chain:** C: **PDB Molecule:**putative membrane protein;<br>**PDBTitle:** the structure of bd oxidase from geobacillus thermodenitrificans<br>**PDB Entry:** PDBe RCSB PDBj
						**PDB header:**dna binding protein

28	c2vqcA_	Alignment	not modelled	6.5	24	**Chain:** A: **PDB Molecule:**hypothetical 13.2 kda protein;<br>**PDBTitle:** structure of a dna binding winged-helix protein, f-112, from2 sulfolobus spindle-shaped virus 1.<br>**PDB Entry:** PDBe RCSB PDBj
29	d2vqca1	Alignment	not modelled	6.5	24	**Fold:** DNA/RNA-binding 3-helical bundle<br>**Superfamily:** "Winged helix" DNA-binding domain<br>**Family:** F112-like<br>**PDB entry:** PDBe RCSB PDBj
30	c7rcvj_	Alignment	not modelled	6.3	21	**PDB header:**photosynthesis<br>**Chain:** J: **PDB Molecule:**photosystem ii reaction center protein j;<br>**PDBTitle:** high-resolution structure of photosystem ii from the mesophilic2 cyanobacterium, synechocystis sp. pcc 6803<br>**PDB Entry:** PDBe RCSB PDBj
31	c5dirB_	Alignment	not modelled	6.0	6	**PDB header:**hydrolase<br>**Chain:** B: **PDB Molecule:**lipoprotein signal peptidase;<br>**PDBTitle:** membrane protein at 2.8 angstroms<br>**PDB Entry:** PDBe RCSB PDBj
32	c2lp1A_	Alignment	not modelled	5.8	8	**PDB header:**membrane protein<br>**Chain:** A: **PDB Molecule:**c99;<br>**PDBTitle:** the solution nmr structure of the transmembrane c-terminal domain of2 the amyloid precursor protein (c99)<br>**PDB Entry:** PDBe RCSB PDBj
33	c6thkA_	Alignment	not modelled	5.8	15	**PDB header:**antimicrobial protein<br>**Chain:** A: **PDB Molecule:**pyocin s5;<br>**PDBTitle:** structural mechanism of pyocin s5 import into pseudomonas aeruginosa<br>**PDB Entry:** PDBe RCSB PDBj
34	d2axtj1	Alignment	not modelled	5.7	7	**Fold:** Single transmembrane helix<br>**Superfamily:** Photosystem II reaction center protein J, PsbJ<br>**Family:** PsbJ-like<br>**PDB entry:** PDBe RCSB PDBj
