# Supplementary material for: Characterization of Virulent T4-Like Acinetobacter baumannii Bacteriophages DLP1 and DLP2
Source: Viruses. 2023 Mar 13;15(3):739. doi: 10.3390/v15030739 (PMC10051250; doi:10.3390/v15030739)
Supplement: Supplementary file 1 [file viruses-15-00739-s001.zip › Supplementary files/SFigure 1_PHYRE2 DLP1 DHFR hit_report.pdf]

Phyre2

Email	dlpeters@ualberta.ca
Description	vB_AbaM_DLP1_-_Dihydrofolate_reductase_protein
Date	Fri Jan 6 04:30:02 GMT 2023
Unique Job ID	eaf44881377889b7

Detailed template information

#	Template	Alignment Coverage	3D Model	Confidence	% i.d.	Template Information
1	d1juva_	Alignment	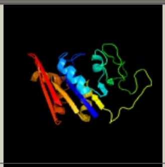	100.0	46	**Fold:** Dihydrofolate reductase-like<br>**Superfamily:** Dihydrofolate reductase-like<br>**Family:** Dihydrofolate reductases<br>**PDB entry:** PDBe RCSB PDBj
2	d1df7a_	Alignment	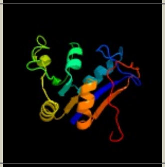	100.0	35	**Fold:** Dihydrofolate reductase-like<br>**Superfamily:** Dihydrofolate reductase-like<br>**Family:** Dihydrofolate reductases<br>**PDB entry:** PDBe RCSB PDBj
3	c2oipE_	Alignment	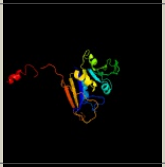	100.0	26	**PDB header:**transferase, oxidoreductase<br>**Chain:** E: **PDB Molecule:**chain a, crystal structure of dhfr;<br>**PDBTitle:** crystal structure of the s290g active site mutant of ts-dhfr from2 cryptosporidium hominis<br>**PDB Entry:** PDBe RCSB PDBj
4	d2fzia1	Alignment	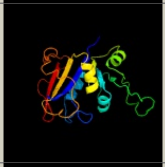	100.0	25	**Fold:** Dihydrofolate reductase-like<br>**Superfamily:** Dihydrofolate reductase-like<br>**Family:** Dihydrofolate reductases<br>**PDB entry:** PDBe RCSB PDBj
5	c3tq8A_	Alignment	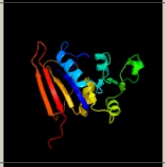	100.0	27	**PDB header:**oxidoreductase/oxidoreductase inhibitor<br>**Chain:** A: **PDB Molecule:**dihydrofolate reductase;<br>**PDBTitle:** structure of the dihydrofolate reductase (fola) from coxiella burnetii2 in complex with trimethoprim<br>**PDB Entry:** PDBe RCSB PDBj
6	d1aoea_	Alignment	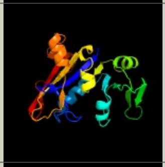	100.0	26	**Fold:** Dihydrofolate reductase-like<br>**Superfamily:** Dihydrofolate reductase-like<br>**Family:** Dihydrofolate reductases<br>**PDB entry:** PDBe RCSB PDBj
7	c3vcoA_	Alignment	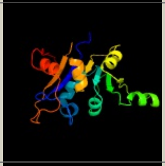	100.0	30	**PDB header:**oxidoreductase<br>**Chain:** A: **PDB Molecule:**dihydrofolate reductase;<br>**PDBTitle:** schistosoma mansoni dihydrofolate reductase<br>**PDB Entry:** PDBe RCSB PDBj
8	c4m7vA_	Alignment	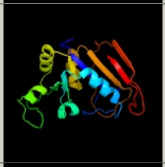	100.0	27	**PDB header:**oxidoreductase<br>**Chain:** A: **PDB Molecule:**dihydrofolate reductase;<br>**PDBTitle:** dihydrofolate reductase from enterococcus faecalis complexed with2 nadp(h)and rab-propyl<br>**PDB Entry:** PDBe RCSB PDBj
9	d8dfra_	Alignment	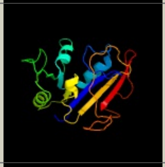	100.0	26	**Fold:** Dihydrofolate reductase-like<br>**Superfamily:** Dihydrofolate reductase-like<br>**Family:** Dihydrofolate reductases<br>**PDB entry:** PDBe RCSB PDBj
10	c7k6cD_	Alignment	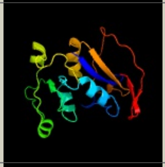	100.0	29	**PDB header:**oxidoreductase/oxidoreductase inhibitor<br>**Chain:** D: **PDB Molecule:**dihydrofolate reductase;<br>**PDBTitle:** crystal structure of dihydrofolate reductase (dhfr) from mycobacterium2 abscessus atcc 19977 / dsm 44196 with nadp and inhibitor p218<br>**PDB Entry:** PDBe RCSB PDBj
11	c4eckB_	Alignment	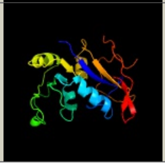	100.0	26	**PDB header:**transferase, oxidoreductase<br>**Chain:** B: **PDB Molecule:**bifunctional dihydrofolate reductase-thymidylate synthase;<br>**PDBTitle:** crystal structure of the toxoplasma gondii ts-dhfr<br>**PDB Entry:** PDBe RCSB PDBj

12	c1zdrB_	Alignment	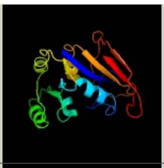	100.0	27	**PDB header:**oxidoreductase<br>**Chain:** B: **PDB Molecule:**dihydrofolate reductase;<br>**PDBTitle:** dhfr from bacillus stearothermophilus<br>**PDB Entry:** PDBe RCSB PDBj
13	d1ra9a_	Alignment	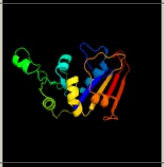	100.0	27	**Fold:** Dihydrofolate reductase-like<br>**Superfamily:** Dihydrofolate reductase-like<br>**Family:** Dihydrofolate reductases<br>**PDB entry:** PDBe RCSB PDBj
14	c6cxmA_	Alignment	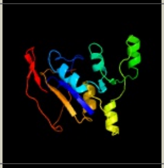	100.0	29	**PDB header:**oxidoreductase/inhibitor<br>**Chain:** A: **PDB Molecule:**dihydrofolate reductase;<br>**PDBTitle:** crystal structure of a dihydrofolate reductase from mycobacterium2 smegmatis in complex with nadp and p218<br>**PDB Entry:** PDBe RCSB PDBj
15	c6drsA_	Alignment	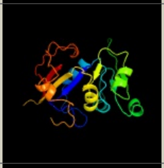	100.0	23	**PDB header:**antifungal protein/inhibitor<br>**Chain:** A: **PDB Molecule:**dihydrofolate reductase, putative;<br>**PDBTitle:** dihydrofolate reductase (dhfr) of aspergillus flavus in complex with a2 small molecule inhibitor<br>**PDB Entry:** PDBe RCSB PDBj
16	c3clbA_	Alignment	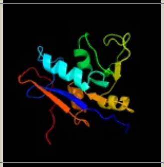	100.0	28	**PDB header:**oxidoreductase, transferase<br>**Chain:** A: **PDB Molecule:**dhfr-ts;<br>**PDBTitle:** structure of bifunctional tcdhfr-ts in complex with tmq<br>**PDB Entry:** PDBe RCSB PDBj
17	c3cseA_	Alignment	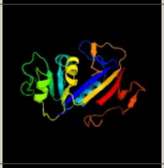	100.0	23	**PDB header:**oxidoreductase<br>**Chain:** A: **PDB Molecule:**dihydrofolate reductase;<br>**PDBTitle:** candida glabrata dihydrofolate reductase complexed with nadph and 2,4-2 diamino-5-(3-(2,5-dimethoxyphenyl)prop-1-ynyl)-6-ethylpyrimidine3 (ucp120b)<br>**PDB Entry:** PDBe RCSB PDBj
18	c7rzoA_	Alignment	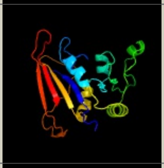	100.0	25	**PDB header:**oxidoreductase<br>**Chain:** A: **PDB Molecule:**dihydrofolate reductase;<br>**PDBTitle:** crystal structure of a dihydrofolate reductase (fola) from2 stenotrophomonas maltophilia<br>**PDB Entry:** PDBe RCSB PDBj
19	c3f0uX_	Alignment	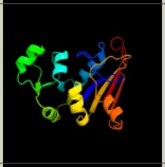	100.0	26	**PDB header:**oxidoreductase<br>**Chain:** X: **PDB Molecule:**trimethoprim-sensitive dihydrofolate reductase;<br>**PDBTitle:** staphylococcus aureus f98y mutant dihydrofolate reductase complexed2 with nadph and 2,4-diamino-5-[3-(3-methoxy-5-phenylphenyl)but-1-3 ynyl]-6-methylpyrimidine<br>**PDB Entry:** PDBe RCSB PDBj
20	c8f09A_	Alignment	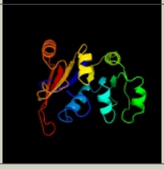	100.0	25	**PDB header:**oxidoreductase<br>**Chain:** A: **PDB Molecule:**dihydrofolate reductase;<br>**PDBTitle:** crystal structure of a trimethoprim-resistant dihydrofolate reductase2 (dhfr) enzyme from an uncultured soil bacterium<br>**PDB Entry:** PDBe RCSB PDBj
21	c3ia5A_	Alignment	not modelled	100.0	25	**PDB header:**oxidoreductase<br>**Chain:** A: **PDB Molecule:**dihydrofolate reductase;<br>**PDBTitle:** moritella profunda dihydrofolate reductase (dhfr)<br>**PDB Entry:** PDBe RCSB PDBj
22	c3e0bA_	Alignment	not modelled	100.0	25	**PDB header:**oxidoreductase<br>**Chain:** A: **PDB Molecule:**dihydrofolate reductase;<br>**PDBTitle:** bacillus anthracis dihydrofolate reductase complexed with nadph and 2,2 4-diamino-5-(3-(2,5-dimethoxyphenyl)prop-1-ynyl)-6-ethylpyrimidine3 (ucp120b)<br>**PDB Entry:** PDBe RCSB PDBj
23	c3ix9B_	Alignment	not modelled	100.0	24	**PDB header:**oxidoreductase<br>**Chain:** B: **PDB Molecule:**dihydrofolate reductase;<br>**PDBTitle:** crystal structure of streptococcus pneumoniae dihydrofolate reductase2 - sp9 mutant<br>**PDB Entry:** PDBe RCSB PDBj
24	d1kmva_	Alignment	not modelled	100.0	23	**Fold:** Dihydrofolate reductase-like<br>**Superfamily:** Dihydrofolate reductase-like<br>**Family:** Dihydrofolate reductases<br>**PDB entry:** PDBe RCSB PDBj
25	d1j3ka_	Alignment	not modelled	100.0	24	**Fold:** Dihydrofolate reductase-like<br>**Superfamily:** Dihydrofolate reductase-like<br>**Family:** Dihydrofolate reductases<br>**PDB entry:** PDBe RCSB PDBj
26	c3dg8B_	Alignment	not modelled	100.0	24	**PDB header:**oxidoreductase, transferase<br>**Chain:** B: **PDB Molecule:**bifunctional dihydrofolate reductase-thymidylate synthase;<br>**PDBTitle:** quadruple mutant (n51i+c59r+s108n+i164l) plasmodium falciparum2 dihydrofolate reductase-thymidylate synthase (pfdhfr-ts) complexed3 with rjf670, nadph, and dump<br>**PDB Entry:** PDBe RCSB PDBj
27	d1vdra_	Alignment	not modelled	100.0	28	**Fold:** Dihydrofolate reductase-like<br>**Superfamily:** Dihydrofolate reductase-like

27	d1vdra_	Alignment	not modelled	100.0	28	**Family:** Dihydrofolate reductases<br>**PDB entry:** PDBe RCSB PDBj
28	c3rg9A_	Alignment	not modelled	100.0	21	**PDB header:**oxidoreductase/oxidoreductase inhibitor<br>**Chain:** A: **PDB Molecule:**bifunctional dihydrofolate reductase-thymidylate synthase;<br>**PDBTitle:** trypanosoma brucei dihydrofolate reductase (tbdhfr) in complex with2 wr99210<br>**PDB Entry:** PDBe RCSB PDBj
29	d1seja1	Alignment	not modelled	100.0	27	**Fold:** Dihydrofolate reductase-like<br>**Superfamily:** Dihydrofolate reductase-like<br>**Family:** Dihydrofolate reductases<br>**PDB entry:** PDBe RCSB PDBj
30	c2blcA_	Alignment	not modelled	100.0	24	**PDB header:**oxidoreductase<br>**Chain:** A: **PDB Molecule:**dihydrofolate reductase-thymidylate synthase;<br>**PDBTitle:** sp21 double mutant p. vivax dihydrofolate reductase in complex with2 des-chloropyrimethamine<br>**PDB Entry:** PDBe RCSB PDBj
31	c5eccA_	Alignment	not modelled	100.0	25	**PDB header:**oxidoreductase/oxidoreductase inhibitor<br>**Chain:** A: **PDB Molecule:**dehydrofolate reductase type i;<br>**PDBTitle:** klebsiella pneumoniae dfra1 complexed with nadph and 6-ethyl-5-(3-(2-2 methoxy-5-(pyridin-4-yl)phenyl)prop-1-yn-1-yl)pyrimidine-2,4-diamine<br>**PDB Entry:** PDBe RCSB PDBj
32	d3dfra_	Alignment	not modelled	100.0	21	**Fold:** Dihydrofolate reductase-like<br>**Superfamily:** Dihydrofolate reductase-like<br>**Family:** Dihydrofolate reductases<br>**PDB entry:** PDBe RCSB PDBj
33	c3k2hA_	Alignment	not modelled	100.0	23	**PDB header:**transferase<br>**Chain:** A: **PDB Molecule:**dihydrofolate reductase/thymidylate synthase;<br>**PDBTitle:** co-crystal structure of dihydrofolate reductase/thymidylate synthase2 from babesia bovis with dump, pemetrexed and nadp<br>**PDB Entry:** PDBe RCSB PDBj
34	c3jsuA_	Alignment	not modelled	100.0	23	**PDB header:**oxidoreductase, transferase<br>**Chain:** A: **PDB Molecule:**dihydrofolate reductase-thymidylate synthase;<br>**PDBTitle:** quadruple mutant(n51i+c59r+s108n+i164l) plasmodium falciparum2 dihydrofolate reductase-thymidylate synthase(pfdhfr-ts) complexed3 with qn254, nadph, and dump<br>**PDB Entry:** PDBe RCSB PDBj
35	d1d1ga_	Alignment	not modelled	99.9	21	**Fold:** Dihydrofolate reductase-like<br>**Superfamily:** Dihydrofolate reductase-like<br>**Family:** Dihydrofolate reductases<br>**PDB entry:** PDBe RCSB PDBj
36	c6rumA_	Alignment	not modelled	100.0	21	**PDB header:**protein binding<br>**Chain:** A: **PDB Molecule:**gfp-lama-g97 a gfp enhancer nanobody with cpdhfr insertion;<br>**PDBTitle:** crystal structure of gfp-lama-g97 - a gfp enhancer nanobody with2 cpdhfr insertion and tmp and nadph<br>**PDB Entry:** PDBe RCSB PDBj
37	c2gd9A_	Alignment	not modelled	99.5	18	**PDB header:**oxidoreductase<br>**Chain:** A: **PDB Molecule:**hypothetical protein yyap;<br>**PDBTitle:** crystal structure of a putative dihydrofolate reductase (bsu40760,2 yyap) from bacillus subtilis at 2.30 a resolution<br>**PDB Entry:** PDBe RCSB PDBj
38	c7lrhD_	Alignment	not modelled	98.2	19	**PDB header:**oxidoreductase<br>**Chain:** D: **PDB Molecule:**riboflavin biosynthesis protein ribd;<br>**PDBTitle:** c-terminal domain of ribd from brucella abortus (5-amino-6-2 ribosylamino-2,4(1h,3h)-pyrimidinedione 5'-phosphate reductase)<br>**PDB Entry:** PDBe RCSB PDBj
39	c2p4gA_	Alignment	not modelled	96.9	18	**PDB header:**oxidoreductase<br>**Chain:** A: **PDB Molecule:**hypothetical protein;<br>**PDBTitle:** crystal structure of a pyrimidine reductase-like protein (dip1392)2 from corynebacterium diphtheriae nctc at 2.30 a resolution<br>**PDB Entry:** PDBe RCSB PDBj
40	c2xw7A_	Alignment	not modelled	99.4	16	**PDB header:**oxidoreductase<br>**Chain:** A: **PDB Molecule:**dihydrofolate reductase;<br>**PDBTitle:** structure of mycobacterium smegmatis putative reductase ms0308<br>**PDB Entry:** PDBe RCSB PDBj
41	c5xuxC_	Alignment	not modelled	97.7	17	**PDB header:**oxidoreductase<br>**Chain:** C: **PDB Molecule:**conserved protein;<br>**PDBTitle:** crystal structure of rib7 from methanosarcina mazei<br>**PDB Entry:** PDBe RCSB PDBj
42	c3kgyA_	Alignment	not modelled	98.4	14	**PDB header:**oxidoreductase<br>**Chain:** A: **PDB Molecule:**bifunctional deaminase-reductase domain protein;<br>**PDBTitle:** crystal structure of putative dihydrofolate reductase (yp_001636057.1)2 from chloroflexus aurantiacus j-10-fl at 1.50 a resolution<br>**PDB Entry:** PDBe RCSB PDBj
43	c2hxvA_	Alignment	not modelled	98.1	15	**PDB header:**biosynthetic protein<br>**Chain:** A: **PDB Molecule:**diaminohydroxyphosphoribosylaminopyrimidine deaminase/ 5-<br>**PDBTitle:** crystal structure of a diaminohydroxyphosphoribosylaminopyrimidine2 deaminase/ 5-amino-6-(5-phosphoribosylamino)uracil reductase (tm1828)3 from thermotoga maritima at 1.80 a resolution<br>**PDB Entry:** PDBe RCSB PDBj
44	d2hxva1	Alignment	not modelled	98.3	17	**Fold:** Dihydrofolate reductase-like<br>**Superfamily:** Dihydrofolate reductase-like<br>**Family:** RibD C-terminal domain-like<br>**PDB entry:** PDBe RCSB PDBj
45	c2o7pA_	Alignment	not modelled	98.3	14	**PDB header:**hydrolase, oxidoreductase<br>**Chain:** A: **PDB Molecule:**riboflavin biosynthesis protein ribd;<br>**PDBTitle:** the crystal structure of ribd from escherichia coli in complex with2 the oxidised nadp+ cofactor in the active site of the reductase3 domain

						**PDB Entry:** PDBe RCSB PDBj
46	c2d5nB_	Alignment	not modelled	98.2	14	**PDB header:**hydrolase, oxidoreductase<br>**Chain:** B: **PDB Molecule:**riboflavin biosynthesis protein ribd;<br>**PDBTitle:** crystal structure of a bifunctional deaminase and reductase2 involved in riboflavin biosynthesis<br>**PDB Entry:** PDBe RCSB PDBj
47	c3jtwB_	Alignment	not modelled	99.5	14	**PDB header:**oxidoreductase<br>**Chain:** B: **PDB Molecule:**dihydrofolate reductase;<br>**PDBTitle:** crystal structure of putative dihydrofolate reductase (yp_805003.1)2 from pediococcus pentosaceus atcc 25745 at 1.90 a resolution<br>**PDB Entry:** PDBe RCSB PDBj
48	d2b3za1	Alignment	not modelled	98.5	15	**Fold:** Dihydrofolate reductase-like<br>**Superfamily:** Dihydrofolate reductase-like<br>**Family:** RibD C-terminal domain-like<br>**PDB entry:** PDBe RCSB PDBj
49	c7lrhC_	Alignment	not modelled	98.2	15	**PDB header:**oxidoreductase<br>**Chain:** C: **PDB Molecule:**riboflavin biosynthesis protein ribd;<br>**PDBTitle:** c-terminal domain of ribd from brucella abortus (5-amino-6-2 ribosylamino-2,4(1h,3h)-pyrimidinedione 5'-phosphate reductase)<br>**PDB Entry:** PDBe RCSB PDBj
50	c3ky8B_	Alignment	not modelled	98.3	13	**PDB header:**biosynthetic protein<br>**Chain:** B: **PDB Molecule:**putative riboflavin biosynthesis protein;<br>**PDBTitle:** crystal structure of putative riboflavin biosynthesis protein2 (yp_001092907.1) from shewanella sp. pv-4 at 2.12 a resolution<br>**PDB Entry:** PDBe RCSB PDBj
51	c6p8cB_	Alignment	not modelled	98.4	14	**PDB header:**oxidoreductase<br>**Chain:** B: **PDB Molecule:**2,5-diamino-6-ribosylamino-4(3h)-pyrimidinone 5'-phosphate<br>**PDBTitle:** 2,5-diamino-6-(ribosylamino)-4(3h)-pyrimidinone 5'-phosphate reductase2 (mthred) from methanothermobacter thermautotrophicus<br>**PDB Entry:** PDBe RCSB PDBj
52	c3zpgA_	Alignment	not modelled	98.0	13	**PDB header:**hydrolase<br>**Chain:** A: **PDB Molecule:**ribd;<br>**PDBTitle:** acinetobacter baumannii ribd, form 2<br>**PDB Entry:** PDBe RCSB PDBj
53	d2azna1	Alignment	not modelled	98.3	14	**Fold:** Dihydrofolate reductase-like<br>**Superfamily:** Dihydrofolate reductase-like<br>**Family:** RibD C-terminal domain-like<br>**PDB entry:** PDBe RCSB PDBj
54	c4xt6A_	Alignment	not modelled	97.2	13	**PDB header:**oxidoreductase<br>**Chain:** A: **PDB Molecule:**rv2671;<br>**PDBTitle:** crystal structure of rv2671 from mycobacterium tuberculosis in complex2 with the tetrahydropteridine ring of tetrahydrofolate (thf)<br>**PDB Entry:** PDBe RCSB PDBj
55	c4cvkA_	Alignment	not modelled	80.6	15	**PDB header:**ligase<br>**Chain:** A: **PDB Molecule:**udp-n-acetylmuramoyl-tripeptide--d-alanyl-d-alanine ligase;<br>**PDBTitle:** pamurf in complex with udp-murnac-tripeptide (mdap)<br>**PDB Entry:** PDBe RCSB PDBj
56	c7waeB_	Alignment	not modelled	77.9	28	**PDB header:**ligase<br>**Chain:** B: **PDB Molecule:**cyanophycin synthase;<br>**PDBTitle:** trichodesmium erythraeum cyanophycin synthetase 1 (tecpha1) with2 atpgammas, 4x(beta-asp-arg), and aspartate<br>**PDB Entry:** PDBe RCSB PDBj
57	c7sy9B_	Alignment	not modelled	64.3	14	**PDB header:**ligase<br>**Chain:** B: **PDB Molecule:**udp-n-acetylmuramoylalanine--d-glutamate ligase;<br>**PDBTitle:** crystal structure of udp-n-acetylmuramoylalanine--d-glutamate ligase2 (murd) from pseudomonas aeruginosa pao1<br>**PDB Entry:** PDBe RCSB PDBj
58	c4ha7B_	Alignment	not modelled	63.1	13	**PDB header:**oxidoreductase<br>**Chain:** B: **PDB Molecule:**2,5-diamino-6-ribosylamino-4(3h)-pyrimidinone 5'-phosphate<br>**PDBTitle:** structural insights into the reduction mechanism of saccharomyces2 cerevisia riboflavin biosynthesis reductase rib7<br>**PDB Entry:** PDBe RCSB PDBj
59	c7lgjC_	Alignment	not modelled	62.8	29	**PDB header:**ligase<br>**Chain:** C: **PDB Molecule:**cyanophycin synthase;<br>**PDBTitle:** cyanophycin synthetase 1 from synechocystis sp. utex2470 with adpcp2 and 8x(asp-arg)-nh2<br>**PDB Entry:** PDBe RCSB PDBj
60	c2xjaD_	Alignment	not modelled	60.1	21	**PDB header:**ligase<br>**Chain:** D: **PDB Molecule:**udp-n-acetylmuramoyl-l-alanyl-d-glutamate--2,6-<br>**PDBTitle:** structure of mure from m.tuberculosis with dipeptide and adp<br>**PDB Entry:** PDBe RCSB PDBj
61	c1j6uA_	Alignment	not modelled	59.2	20	**PDB header:**ligase<br>**Chain:** A: **PDB Molecule:**udp-n-acetylmuramate-alanine ligase murc;<br>**PDBTitle:** crystal structure of udp-n-acetylmuramate-alanine ligase murc (tm0231)2 from thermotoga maritima at 2.3 a resolution<br>**PDB Entry:** PDBe RCSB PDBj
62	c3uhjE_	Alignment	not modelled	58.1	15	**PDB header:**oxidoreductase<br>**Chain:** E: **PDB Molecule:**probable glycerol dehydrogenase;<br>**PDBTitle:** crystal structure of a probable glycerol dehydrogenase from2 sinorhizobium meliloti 1021<br>**PDB Entry:** PDBe RCSB PDBj
63	c6vr7A_	Alignment	not modelled	56.9	17	**PDB header:**ligase<br>**Chain:** A: **PDB Molecule:**mur ligase middle domain protein;<br>**PDBTitle:** structure of a pseudomurein peptide ligase type c from methanothermus2 fervidus<br>**PDB Entry:** PDBe RCSB PDBj
64	c3zdrA_	Alignment	not modelled	56.6	12	**PDB header:**oxidoreductase<br>**Chain:** A: **PDB Molecule:**alcohol dehydrogenase domain of the bifunctional<br>**PDBTitle:** structure of the alcohol dehydrogenase (adh) domain of a2 bifunctional adhe dehydrogenase from geobacillus3

						thermoglucosidasius ncimb 11955<br>**PDB Entry:** PDBe RCSB PDBj
65	c4rflB_	Alignment	not modelled	54.8	19	**PDB header:**oxidoreductase<br>**Chain:** B: **PDB Molecule:**glycerol-1-phosphate dehydrogenase [nad(p)+];<br>**PDBTitle:** crystal structure of g1pdh with nadph from methanocaldococcus2 jannaschii<br>**PDB Entry:** PDBe RCSB PDBj
66	c4c13A_	Alignment	not modelled	47.0	14	**PDB header:**ligase<br>**Chain:** A: **PDB Molecule:**udp-n-acetylmuramoyl-l-alanyl-d-glutamate--l-lysine ligase;<br>**PDBTitle:** x-ray crystal structure of staphylococcus aureus mure with udp-murnac-2 ala-glu-lys<br>**PDB Entry:** PDBe RCSB PDBj
67	c7lgnB_	Alignment	not modelled	46.5	19	**PDB header:**ligase<br>**Chain:** B: **PDB Molecule:**cyanophycin synthase;<br>**PDBTitle:** cyanophycin synthetase 1 from t. morbirosei<br>**PDB Entry:** PDBe RCSB PDBj
68	c7bvaB_	Alignment	not modelled	42.1	11	**PDB header:**ligase<br>**Chain:** B: **PDB Molecule:**udp-n-acetylmuramate--l-alanine ligase;<br>**PDBTitle:** crystal structure of udp-n-acetylmuramic acid l-alanine ligase (murc)2 from mycobacterium bovis<br>**PDB Entry:** PDBe RCSB PDBj
69	c1e8cB_	Alignment	not modelled	41.9	23	**PDB header:**ligase<br>**Chain:** B: **PDB Molecule:**udp-n-acetylmuramoylalanyl-d-glutamate--2,6-diaminopimelate<br>**PDBTitle:** structure of mure the udp-n-acetylmuramyl tripeptide synthetase from2 e. coli<br>**PDB Entry:** PDBe RCSB PDBj
70	d1gg4a1	Alignment	not modelled	39.9	15	**Fold:** MurD-like peptide ligases, peptide-binding domain<br>**Superfamily:** MurD-like peptide ligases, peptide-binding domain<br>**Family:** MurCDEF C-terminal domain<br>**PDB entry:** PDBe RCSB PDBj
71	c6csjD_	Alignment	not modelled	38.9	24	**PDB header:**oxidoreductase<br>**Chain:** D: **PDB Molecule:**glycerol dehydrogenase;<br>**PDBTitle:** structure of a bacillus coagulans polyol dehydrogenase double mutant2 with an acquired d-lactate dehydrogenase activity<br>**PDB Entry:** PDBe RCSB PDBj
72	c6emvA_	Alignment	not modelled	36.1	20	**PDB header:**rna binding protein<br>**Chain:** A: **PDB Molecule:**trna (guanine(9)-/adenine(9)-n1)-methyltransferase;<br>**PDBTitle:** crystal structure of dual specific trm10 construct from thermococcus2 kodakaraensis.<br>**PDB Entry:** PDBe RCSB PDBj
73	c7d27A_	Alignment	not modelled	35.9	31	**PDB header:**ligase<br>**Chain:** A: **PDB Molecule:**udp-n-acetylmuramoyl-l-alanyl-d-glutamate--2,6-<br>**PDBTitle:** structure of udp-n-acetylmuramoyl-l-alanyl-d-glutamate--2, 6-2 diaminopimelate ligase<br>**PDB Entry:** PDBe RCSB PDBj
74	c1gqqA_	Alignment	not modelled	34.9	4	**PDB header:**cell wall biosynthesis<br>**Chain:** A: **PDB Molecule:**udp-n-acetylmuramate-l-alanine ligase;<br>**PDBTitle:** murc - crystal structure of the apo-enzyme from haemophilus influenzae<br>**PDB Entry:** PDBe RCSB PDBj
75	c3ox4D_	Alignment	not modelled	34.8	15	**PDB header:**oxidoreductase<br>**Chain:** D: **PDB Molecule:**alcohol dehydrogenase 2;<br>**PDBTitle:** structures of iron-dependent alcohol dehydrogenase 2 from zymomonas2 mobilis zm4 complexed with nad cofactor<br>**PDB Entry:** PDBe RCSB PDBj
76	c1o5zA_	Alignment	not modelled	33.5	23	**PDB header:**ligase<br>**Chain:** A: **PDB Molecule:**folylpolyglutamate synthase/dihydrofolate synthase;<br>**PDBTitle:** crystal structure of folylpolyglutamate synthase (tm0166) from2 thermotoga maritima at 2.10 a resolution<br>**PDB Entry:** PDBe RCSB PDBj
77	c2dzaA_	Alignment	not modelled	32.5	18	**PDB header:**transferase<br>**Chain:** A: **PDB Molecule:**dihydropteroate synthase;<br>**PDBTitle:** crystal structure of dihydropteroate synthase from thermus2 thermophilus hb8 in complex with 4-aminobenzoate<br>**PDB Entry:** PDBe RCSB PDBj
78	c5fb3C_	Alignment	not modelled	30.9	12	**PDB header:**oxidoreductase<br>**Chain:** C: **PDB Molecule:**glycerol-1-phosphate dehydrogenase [nad(p)+];<br>**PDBTitle:** structure of glycerophosphate dehydrogenase in complex with nadph<br>**PDB Entry:** PDBe RCSB PDBj
79	c6istA_	Alignment	not modelled	29.8	31	**PDB header:**hydrolase<br>**Chain:** A: **PDB Molecule:**lysin;<br>**PDBTitle:** crystal structure of a wild type endolysin lysime-ef1<br>**PDB Entry:** PDBe RCSB PDBj
80	c2f00A_	Alignment	not modelled	29.7	8	**PDB header:**ligase<br>**Chain:** A: **PDB Molecule:**udp-n-acetylmuramate--l-alanine ligase;<br>**PDBTitle:** escherichia coli murc<br>**PDB Entry:** PDBe RCSB PDBj
81	c5zwoY_	Alignment	not modelled	29.1	27	**PDB header:**splicing<br>**Chain:** Y: **PDB Molecule:**pre-mrna-splicing factor cwc26;<br>**PDBTitle:** cryo-em structure of the yeast b complex at average resolution of 3.92 angstrom<br>**PDB Entry:** PDBe RCSB PDBj
82	c3hl0B_	Alignment	not modelled	28.5	15	**PDB header:**oxidoreductase<br>**Chain:** B: **PDB Molecule:**maleylacetate reductase;<br>**PDBTitle:** crystal structure of maleylacetate reductase from agrobacterium2 tumefaciens<br>**PDB Entry:** PDBe RCSB PDBj
83	d1o6da_	Alignment	not modelled	28.3	24	**Fold:** alpha/beta knot<br>**Superfamily:** alpha/beta knot<br>**Family:** YbeA-like<br>**PDB entry:** PDBe RCSB PDBj
84	c1xahA_	Alignment	not modelled	27.4	15	**PDB header:**lyase<br>**Chain:** A: **PDB Molecule:**3-dehydroquinate synthase;<br>**PDBTitle:** crystal structure of staphlyococcus aureus 3-dehydroquinate2 synthase (dhqs) in complex with zn2+ and nad+

						**PDB Entry:** PDBe RCSB PDBj
85	d2gc6a1	Alignment	not modelled	24.2	22	**Fold:** MurD-like peptide ligases, peptide-binding domain<br>**Superfamily:** MurD-like peptide ligases, peptide-binding domain<br>**Family:** Folylpolyglutamate synthetase, C-terminal domain<br>**PDB entry:** PDBe RCSB PDBj
86	d1oj7a_	Alignment	not modelled	21.1	15	**Fold:** Dehydroquinate synthase-like<br>**Superfamily:** Dehydroquinate synthase-like<br>**Family:** Iron-containing alcohol dehydrogenase<br>**PDB entry:** PDBe RCSB PDBj
87	c1ta9A_	Alignment	not modelled	20.0	15	**PDB header:**oxidoreductase<br>**Chain:** A: **PDB Molecule:**glycerol dehydrogenase;<br>**PDBTitle:** crystal structure of glycerol dehydrogenase from schizosaccharomyces2 pombe<br>**PDB Entry:** PDBe RCSB PDBj
88	c5mtzA_	Alignment	not modelled	19.2	17	**PDB header:**hydrolase<br>**Chain:** A: **PDB Molecule:**ribonuclease z;<br>**PDBTitle:** crystal structure of a long form rnase z from yeast<br>**PDB Entry:** PDBe RCSB PDBj
89	d1vh0a_	Alignment	not modelled	18.4	24	**Fold:** alpha/beta knot<br>**Superfamily:** alpha/beta knot<br>**Family:** YbeA-like<br>**PDB entry:** PDBe RCSB PDBj
90	d1j6ua2	Alignment	not modelled	18.3	14	**Fold:** MurD-like peptide ligases, peptide-binding domain<br>**Superfamily:** MurD-like peptide ligases, peptide-binding domain<br>**Family:** MurCDEF C-terminal domain<br>**PDB entry:** PDBe RCSB PDBj
91	c5uurA_	Alignment	not modelled	18.2	18	**PDB header:**transferase<br>**Chain:** A: **PDB Molecule:**dihydropteroate synthase;<br>**PDBTitle:** xanthomonas albilineans dihydropteroate synthase with 4-aminobenzoic2 acid<br>**PDB Entry:** PDBe RCSB PDBj
92	c5okzJ_	Alignment	not modelled	18.0	50	**PDB header:**rna binding protein<br>**Chain:** J: **PDB Molecule:**m-phase phosphoprotein 6 homolog;<br>**PDBTitle:** crystal strucrure of the mpp6 exosome complex<br>**PDB Entry:** PDBe RCSB PDBj
93	d1jq5a_	Alignment	not modelled	17.4	19	**Fold:** Dehydroquinate synthase-like<br>**Superfamily:** Dehydroquinate synthase-like<br>**Family:** Iron-containing alcohol dehydrogenase<br>**PDB entry:** PDBe RCSB PDBj
94	c3uagA_	Alignment	not modelled	17.3	18	**PDB header:**ligase<br>**Chain:** A: **PDB Molecule:**protein (udp-n-acetylmuramoyl-l-alanine:d-glutamate<br>**PDBTitle:** udp-n-acetylmuramoyl-l-alanine:d-glutamate ligase<br>**PDB Entry:** PDBe RCSB PDBj
95	c3en2A_	Alignment	not modelled	16.7	6	**PDB header:**structural genomics, unknown function<br>**Chain:** A: **PDB Molecule:**probable primosomal replication protein n;<br>**PDBTitle:** three-dimensional structure of the protein prib from ralstonia2 solanacearum at the resolution 2.3a. northeast structural genomics3 consortium target rsr213c.<br>**PDB Entry:** PDBe RCSB PDBj
96	c5tprB_	Alignment	not modelled	15.7	12	**PDB header:**lyase<br>**Chain:** B: **PDB Molecule:**3-dehydroquinate synthase;<br>**PDBTitle:** desmethyl-4-deoxygadusol synthase from anabaena variabilis (ava_3858)2 with nad+ and zn2+ bound<br>**PDB Entry:** PDBe RCSB PDBj
97	c7w9yB_	Alignment	not modelled	14.9	11	**PDB header:**oxidoreductase<br>**Chain:** B: **PDB Molecule:**iron-containing alcohol dehydrogenase;<br>**PDBTitle:** crystal structure of bacillus subtilis yugj in complex with nadp and2 nickel<br>**PDB Entry:** PDBe RCSB PDBj
98	c2gruB_	Alignment	not modelled	14.9	16	**PDB header:**lyase<br>**Chain:** B: **PDB Molecule:**2-deoxy-scyllo-inosose synthase;<br>**PDBTitle:** crystal structure of 2-deoxy-scyllo-inosose synthase2 complexed with carbaglucose-6-phosphate, nad+ and co2+<br>**PDB Entry:** PDBe RCSB PDBj
99	c1gg4A_	Alignment	not modelled	14.8	16	**PDB header:**ligase<br>**Chain:** A: **PDB Molecule:**udp-n-acetylmuramoylalanyl-d-glutamyl-2,6-diaminopimelate-<br>**PDBTitle:** crystal structure of escherichia coli udpmurnac-tripeptide d-alanyl-d-2 alanine-adding enzyme (murf) at 2.3 angstrom resolution<br>**PDB Entry:** PDBe RCSB PDBj
