# Supplementary material for: Characterization of Virulent T4-Like Acinetobacter baumannii Bacteriophages DLP1 and DLP2
Source: Viruses. 2023 Mar 13;15(3):739. doi: 10.3390/v15030739 (PMC10051250; doi:10.3390/v15030739)
Supplement: Supplementary file 1 [file viruses-15-00739-s001.zip › Supplementary files/SFigure 5_PHYRE2 DLP1 gp008 hit_report.pdf]

Phyre2

Email	dlpeters@ualberta.ca
Description	SB1_-_ADLP1_008_translation_Acinetobacter_phage_SB1,_complete_genome
Date	Fri Jan 6 04:30:02 GMT 2023
Unique Job ID	8f19ade380738c76

Detailed template information

#	Template	Alignment Coverage	3D Model	Confidence	% i.d.	Template Information
1	d1s7ba_	Alignment	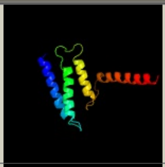	100.0	32	**Fold:** Multidrug resistance efflux transporter EmrE<br>**Superfamily:** Multidrug resistance efflux transporter EmrE<br>**Family:** Multidrug resistance efflux transporter EmrE<br>**PDB entry:** PDBe RCSB PDBj
2	c2i68B_	Alignment	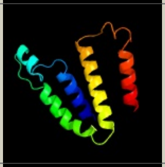	99.9	32	**PDB header:**transport protein<br>**Chain:** B: **PDB Molecule:**protein emre;<br>**PDBTitle:** cryo-em based theoretical model structure of transmembrane2 domain of the multidrug-resistance antiporter from e. coli3 emre<br>**PDB Entry:** PDBe RCSB PDBj
3	c6wk9B_	Alignment	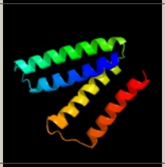	100.0	22	**PDB header:**transport protein<br>**Chain:** B: **PDB Molecule:**multidrug resistance protein, smr family;<br>**PDBTitle:** crystal structure of gdx-clo from small multidrug resistance family of2 transporters in complex with octylguanidinium<br>**PDB Entry:** PDBe RCSB PDBj
4	c7pafA_	Alignment	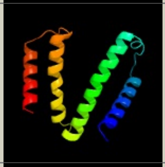	97.8	15	**PDB header:**transport protein<br>**Chain:** A: **PDB Molecule:**licb protein;<br>**PDBTitle:** streptococcus pneumoniae choline importer licb in lipid nanodiscs<br>**PDB Entry:** PDBe RCSB PDBj
5	c6ukjA_	Alignment	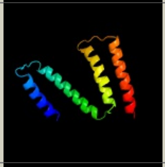	97.7	15	**PDB header:**membrane protein<br>**Chain:** A: **PDB Molecule:**chloroquine resistance transporter;<br>**PDBTitle:** single-particle cryo-em structure of plasmodium falciparum chloroquine2 resistance transporter (pfcrt) 7g8 isoform<br>**PDB Entry:** PDBe RCSB PDBj
6	c5i20E_	Alignment	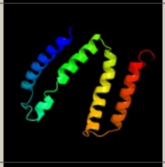	98.2	14	**PDB header:**membrane protein<br>**Chain:** E: **PDB Molecule:**uncharacterized protein;<br>**PDBTitle:** crystal structure of protein<br>**PDB Entry:** PDBe RCSB PDBj
7	c5y79A_	Alignment	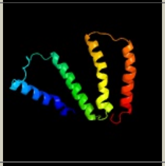	97.9	13	**PDB header:**transport protein<br>**Chain:** A: **PDB Molecule:**putative hexose phosphate translocator;<br>**PDBTitle:** crystal structure of the triose-phosphate/phosphate translocator in2 complex with 3-phosphoglycerate<br>**PDB Entry:** PDBe RCSB PDBj
8	c5ogeE_	Alignment	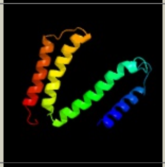	97.8	12	**PDB header:**membrane protein<br>**Chain:** E: **PDB Molecule:**gdp-mannose transporter 1;<br>**PDBTitle:** crystal structure of a nucleotide sugar transporter<br>**PDB Entry:** PDBe RCSB PDBj
9	c5i20C_	Alignment	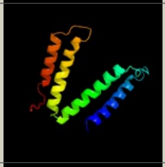	97.8	11	**PDB header:**membrane protein<br>**Chain:** C: **PDB Molecule:**uncharacterized protein;<br>**PDBTitle:** crystal structure of protein<br>**PDB Entry:** PDBe RCSB PDBj
10	c6i1rA_	Alignment	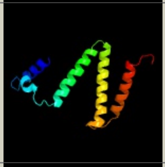	98.1	10	**PDB header:**membrane protein<br>**Chain:** A: **PDB Molecule:**cmp-sialic acid transporter 1;<br>**PDBTitle:** crystal structure of cmp bound cst in an outward facing conformation<br>**PDB Entry:** PDBe RCSB PDBj
11	c6oh2A_	Alignment	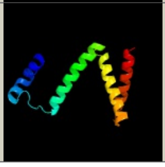	98.2	7	**PDB header:**transport protein<br>**Chain:** A: **PDB Molecule:**cmp-sialic acid transporter;<br>**PDBTitle:** x-ray crystal structure of the mouse cmp-sialic acid transporter in2 complex with cmp, by lipidic cubic phase<br>**PDB Entry:** PDBe RCSB PDBj

12	c6l85A_	Alignment	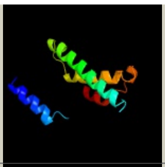	18.5	7	**PDB header:**membrane protein<br>**Chain:** A: **PDB Molecule:**phosphate transporter;<br>**PDBTitle:** the sodium-dependent phosphate transporter<br>**PDB Entry:** PDBe RCSB PDBj
13	c4oo9A_	Alignment	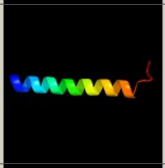	16.1	26	**PDB header:**membrane protein<br>**Chain:** A: **PDB Molecule:**metabotropic glutamate receptor 5, lysozyme, metabotropic<br>**PDBTitle:** structure of the human class c gpcr metabotropic glutamate receptor 52 transmembrane domain in complex with the negative allosteric3 modulator mavoglurant<br>**PDB Entry:** PDBe RCSB PDBj
14	c6rfsX_	Alignment	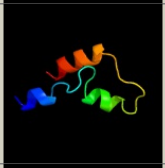	15.9	21	**PDB header:**oxidoreductase<br>**Chain:** X: **PDB Molecule:**subunit nuxm of nadh:ubiquinone oxidoreductase (complex i);<br>**PDBTitle:** cryo-em structure of a respiratory complex i mutant lacking ndufs4<br>**PDB Entry:** PDBe RCSB PDBj
15	c6yp7j_	Alignment	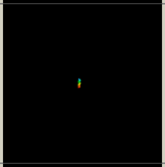	9.4	7	**PDB header:**photosynthesis<br>**Chain:** J: **PDB Molecule:**photosystem ii reaction center protein j;<br>**PDBTitle:** psii-lhcii c2s2 supercomplex from pisum sativum grown in high light2 conditions<br>**PDB Entry:** PDBe RCSB PDBj
16	c6yp7J_	Alignment	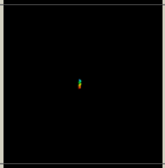	9.4	7	**PDB header:**photosynthesis<br>**Chain:** J: **PDB Molecule:**photosystem ii reaction center protein j;<br>**PDBTitle:** psii-lhcii c2s2 supercomplex from pisum sativum grown in high light2 conditions<br>**PDB Entry:** PDBe RCSB PDBj
17	c5xnmj_	Alignment	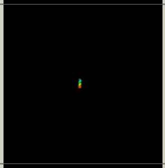	9.3	7	**PDB header:**membrane protein<br>**Chain:** J: **PDB Molecule:**photosystem ii reaction center protein j;<br>**PDBTitle:** structure of unstacked c2s2m2-type psii-lhcii supercomplex from pisum2 sativum<br>**PDB Entry:** PDBe RCSB PDBj
18	c7dtvA_	Alignment	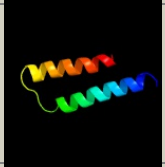	9.2	20	**PDB header:**membrane protein<br>**Chain:** A: **PDB Molecule:**extracellular calcium-sensing receptor;<br>**PDBTitle:** human calcium-sensing receptor bound with l-trp and calcium ions<br>**PDB Entry:** PDBe RCSB PDBj
19	c3jcuj_	Alignment	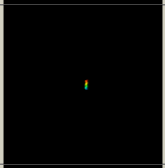	8.6	7	**PDB header:**membrane protein<br>**Chain:** J: **PDB Molecule:**photosystem ii reaction center protein j;<br>**PDBTitle:** cryo-em structure of spinach psii-lhcii supercomplex at 3.2 angstrom2 resolution<br>**PDB Entry:** PDBe RCSB PDBj
20	c7shfB_	Alignment	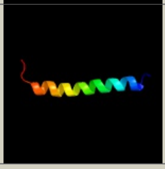	8.0	3	**PDB header:**signaling protein<br>**Chain:** B: **PDB Molecule:**g-protein coupled receptor 158;<br>**PDBTitle:** cryo-em structure of gpr158 coupled to the rgs7-gbeta5 complex<br>**PDB Entry:** PDBe RCSB PDBj
21	c2lp1A_	Alignment	not modelled	7.1	8	**PDB header:**membrane protein<br>**Chain:** A: **PDB Molecule:**c99;<br>**PDBTitle:** the solution nmr structure of the transmembrane c-terminal domain of2 the amyloid precursor protein (c99)<br>**PDB Entry:** PDBe RCSB PDBj
22	c7vcfK_	Alignment	not modelled	6.6	18	**PDB header:**translocase<br>**Chain:** K: **PDB Molecule:**toc10;<br>**PDBTitle:** cryo-em structure of chlamydomonas toc-tic supercomplex<br>**PDB Entry:** PDBe RCSB PDBj
23	c5ir6C_	Alignment	not modelled	6.6	17	**PDB header:**oxidoreductase<br>**Chain:** C: **PDB Molecule:**putative membrane protein;<br>**PDBTitle:** the structure of bd oxidase from geobacillus thermodenitrificans<br>**PDB Entry:** PDBe RCSB PDBj
24	c5doqC_	Alignment	not modelled	6.6	17	**PDB header:**oxidoreductase<br>**Chain:** C: **PDB Molecule:**putative membrane protein;<br>**PDBTitle:** the structure of bd oxidase from geobacillus thermodenitrificans<br>**PDB Entry:** PDBe RCSB PDBj
25	c3mp7B_	Alignment	not modelled	6.6	29	**PDB header:**protein transport<br>**Chain:** B: **PDB Molecule:**preprotein translocase subunit sece;<br>**PDBTitle:** lateral opening of a translocon upon entry of protein suggests the2 mechanism of insertion into membranes<br>**PDB Entry:** PDBe RCSB PDBj
26	c6thkA_	Alignment	not modelled	5.8	15	**PDB header:**antimicrobial protein<br>**Chain:** A: **PDB Molecule:**pyocin s5;<br>**PDBTitle:** structural mechanism of pyocin s5 import into pseudomonas aeruginosa<br>**PDB Entry:** PDBe RCSB PDBj
						**PDB header:**photosynthesis<br>**Chain:** J: **PDB Molecule:**photosystem ii reaction center protein j;

27	c7rcvj_	Alignment	not modelled	5.7	21	**PDBTitle:** high-resolution structure of photosystem ii from the mesophilic2 cyanobacterium, synechocystis sp. pcc 6803<br>**PDB Entry:** PDBe RCSB PDBj
28	d2axtj1	Alignment	not modelled	5.6	7	**Fold:** Single transmembrane helix<br>**Superfamily:** Photosystem II reaction center protein J, PsbJ<br>**Family:** PsbJ-like<br>**PDB entry:** PDBe RCSB PDBj
29	c3a0hJ_	Alignment	not modelled	5.6	7	**PDB header:**electron transport<br>**Chain:** J: **PDB Molecule:**photosystem ii reaction center protein j;<br>**PDBTitle:** crystal structure of i-substituted photosystem ii complex<br>**PDB Entry:** PDBe RCSB PDBj
