# Supplementary figures and images for: Characterization of Virulent T4-Like Acinetobacter baumannii Bacteriophages DLP1 and DLP2
Source: Viruses. 2023 Mar 13;15(3):739. doi: 10.3390/v15030739 (PMC10051250; doi:10.3390/v15030739)

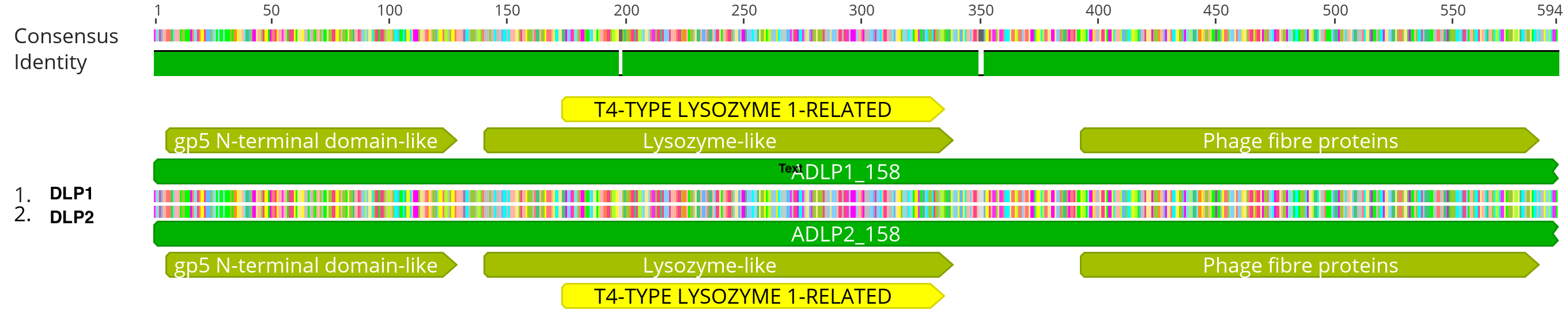

Supplement: Supplementary file 1 [file viruses-15-00739-s001.zip › Supplementary files/SFigure 11_DLP1 DLP2 LTF OMEGA AA alignment.png]

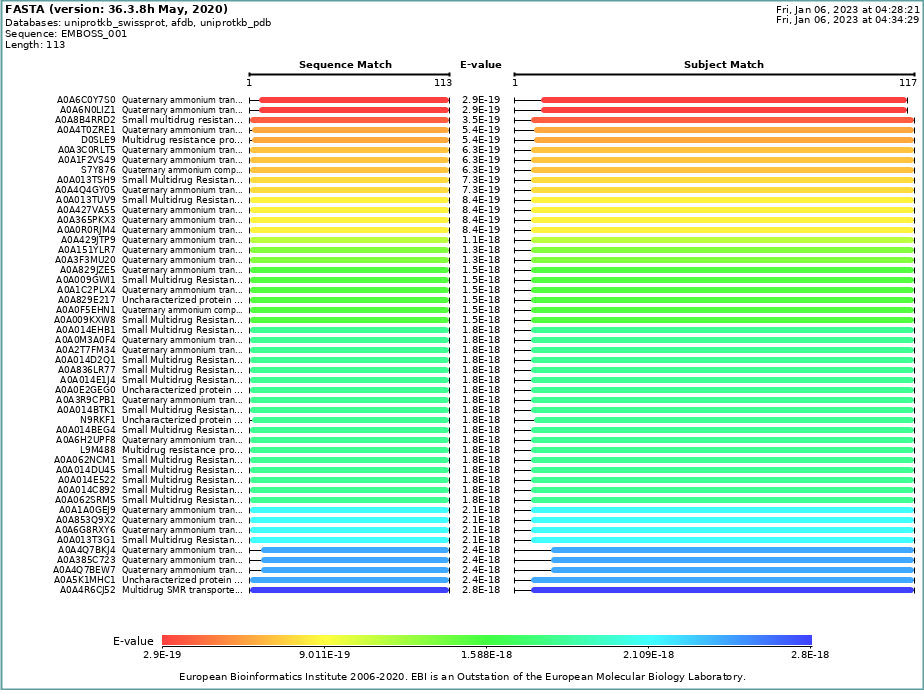

Supplement: Supplementary file 1 [file viruses-15-00739-s001.zip › Supplementary files/SFigure 7_DLP1_gp008.png]

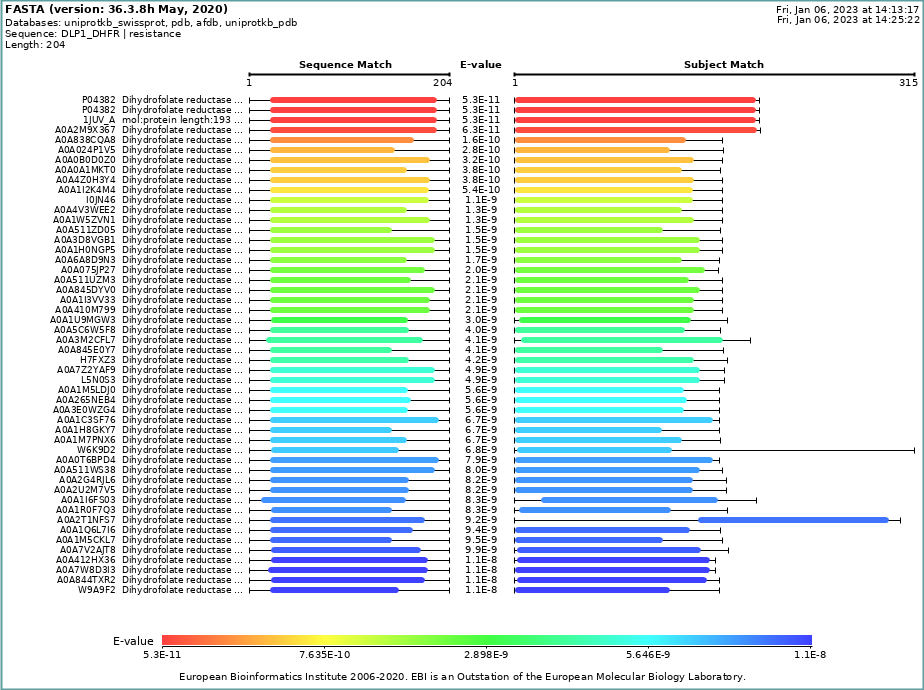

Supplement: Supplementary file 1 [file viruses-15-00739-s001.zip › Supplementary files/SFigure 3_DLP1_DHFR_EBI.png]

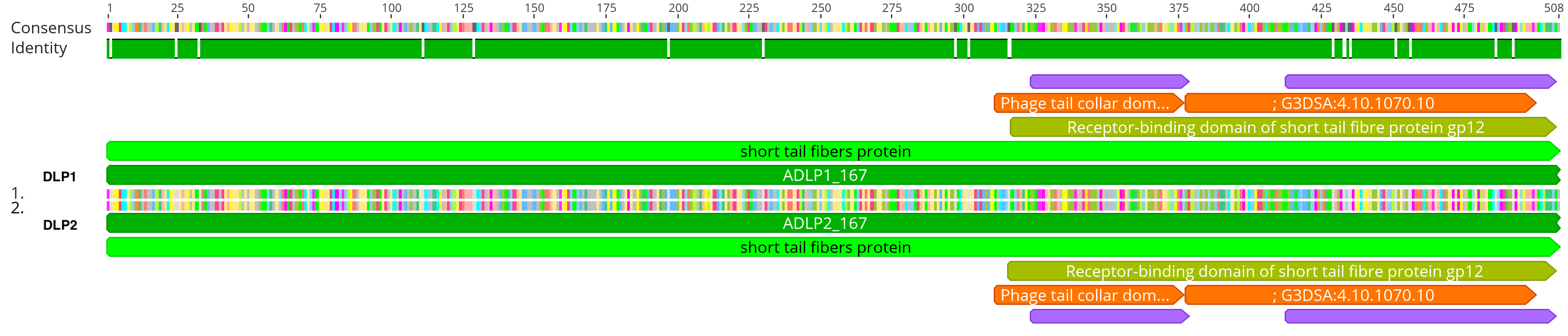

Supplement: Supplementary file 1 [file viruses-15-00739-s001.zip › Supplementary files/SFigure 10_DLP1 DLP2 STF OMEGA AA alignment.png]

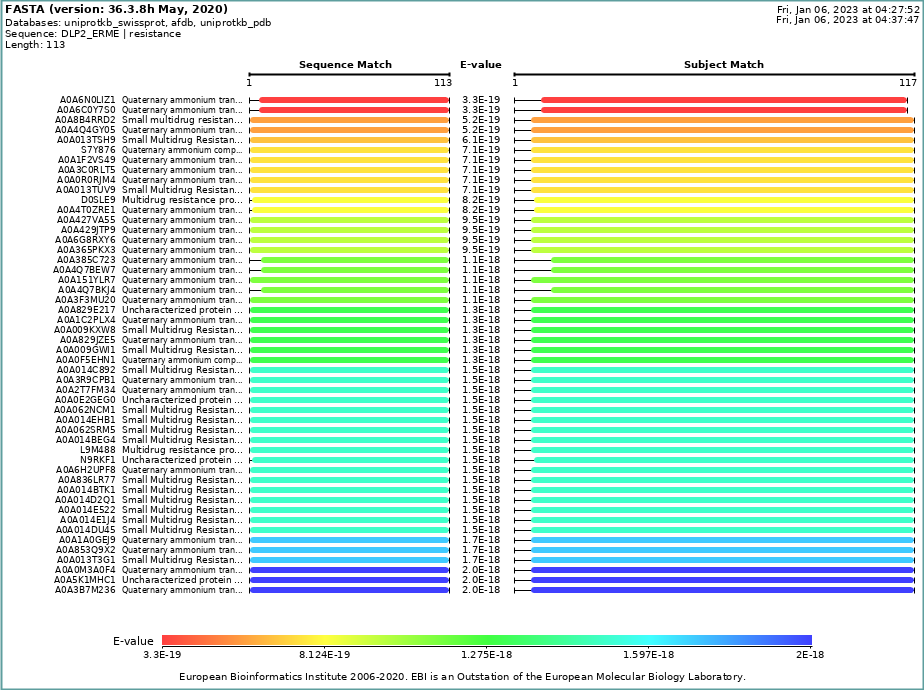

Supplement: Supplementary file 1 [file viruses-15-00739-s001.zip › Supplementary files/SFigure 8_DLP2_gp008.png]

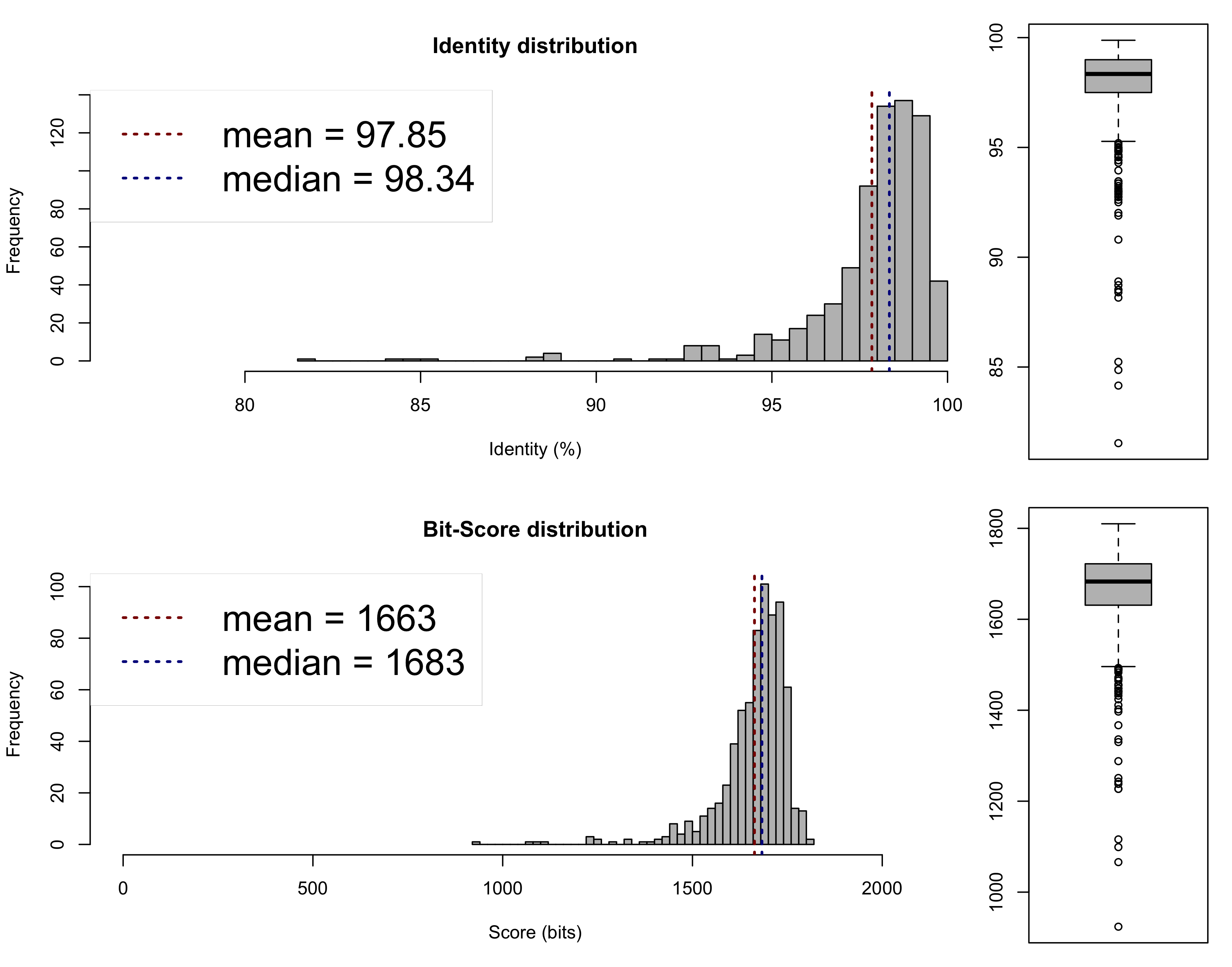

Supplement: Supplementary file 1 [file viruses-15-00739-s001.zip › Supplementary files/SFigure 9_ANI.png]

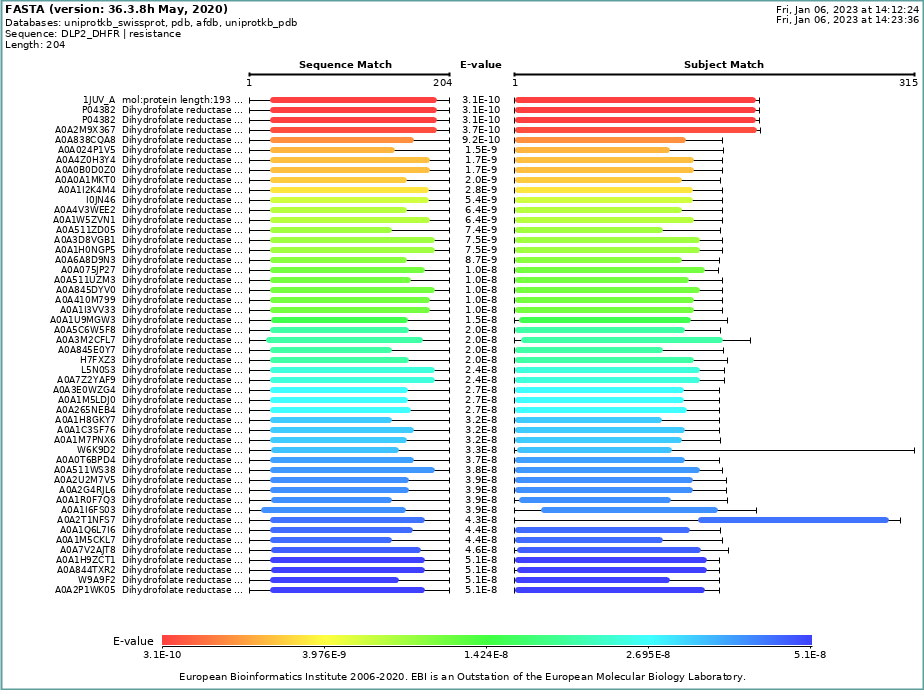

Supplement: Supplementary file 1 [file viruses-15-00739-s001.zip › Supplementary files/SFigure 4_DLP2_DHFR_EBI.png]
